# Exogenous HMGB1 Promotes the Proliferation and Metastasis of Pancreatic Cancer Cells

**DOI:** 10.3389/fmed.2021.756988

**Published:** 2021-11-03

**Authors:** Li Zhu, Shuai Ren, Marcus J. Daniels, Wenli Qiu, Lian Song, Tao You, Dongqing Wang, Zhongqiu Wang

**Affiliations:** ^1^Department of Radiology, Jiangsu Province Hospital of Chinese Medicine, Affiliated Hospital of Nanjing University of Chinese Medicine, Nanjing, China; ^2^Department of Radiology, Johns Hopkins University School of Medicine, Baltimore, MD, United States; ^3^Department of Radiology, Affiliated Hospital of Jiangsu University, Zhenjiang, China; ^4^Department of Radiotherapy, Affiliated Hospital of Jiangsu University, Zhenjiang, China

**Keywords:** exogenous HMGB1, pancreatic cancer, proliferation, metastasis, radiotherapy

## Abstract

**Background:** Exogenous HMGB1 plays a vital role in tumor recurrence, and HMGB1 is ubiquitous in the tumor microenvironment. However, the mechanism of action is still unclear. We investigated the role of exogenous HMGB1 in tumor proliferation and metastasis using human SW1990 and PANC-1 cells after radiotherapy and explored the possible molecular mechanism.

**Materials and Methods:** Residual PANC-1 cells and SW1990 cells were isolated after radiotherapy. The supernatant after radiotherapy was collected. The relative expression of HMGB1 was evaluated by Enzyme Linked Immunosorbent Assay (ELISA). Electron microscope (EMS) was used to collect the images of pancreatic cancer cells pre and post radiotherapy treatment. The proliferation of pancreatic cancer cells which were treated with different radiation doses was measured by Carboxy Fluorescein Succinimidyl Ester (CFSE). The migration rates of pancreatic cancer cells were measured by wound healing assays. Subsequently, the expression of related proteins was detected by Western Blot. *In vivo*, the subcutaneous pancreatic tumor models of nude mice were established, and therapeutic capabilities were tested.

**Results:** HMGB1 was detected in the supernatant of pancreatic cancer cells after radiotherapy. The results of CFSE showed that exogenous HMGB1 promotes the proliferation and metastasis of pancreatic cancer cells. The western blot results showed activation of p-GSK 3β and up-regulation of N-CA, Bcl-2, and Ki67 in response to HMGB1 stimulation, while E-CA expression was down-regulated in pancreatic cancer cells in response to HMGB1 stimulation. *In vivo*, ethyl pyruvate (EP, HMGB1 inhibitor) inhibits the growth of tumors and HMGB1 promotes the proliferation of tumors after radiation.

**Conclusion:** Radiotherapy induces HMGB1 release into the extracellular space. Exogenous HMGB1 promotes the proliferation and metastasis of PANC-1 cells and SW1990 cells by activation of p-GSK 3β which is mediated by Wnt pathway.

## Background

Pancreatic cancer (PC) is a highly fatal malignancy and is the seventh leading cause of cancer death. The incidence and mortality rates of PC remain high in both males and females (1). Due to the insidious onset, rapid development, and poor prognosis, PC is a serious threat to human health. The 5-year survival rate of PC is <5%, giving PC patients the poorest prognosis among those suffering from malignant cancers (2). Unfortunately, although we have made progress in the identification and management of other solid organ tumors, the diagnostic rate of PC has been increasing over the last 10 years likely due to the fact that PC possesses the ability to resist conventional diagnostic agents. No biomarker, neither alone nor in combination, has been superior to carbohydrate antigen 19–9 (CA19–9) in sensitivity and specificity in detecting PC (3). Unfortunately, even with timely diagnosis, <20% of patients with PC are surgical candidates; however many patients could be treated with chemotherapy and/or radiotherapy (4). Even with surgical resection, PC patients still have a poor prognosis with incidence of cancer recurrence rate ranges between 20% and 60%. These findings highlight the importance of identifying the pathogenesis pathways and developing therapies to treat PC.

Cancer radiation therapy is one of the main cancer treatment strategies, and more than half of cancer patients receive radiation therapy to cure local disease, reduce symptoms, or control the disease (5). PC remains one of the deadliest malignancies due to limited treatment options and resistance to radiotherapy (6). Radiation resistance has become a hot topic in the world (7). Some studies have shown that the radio-sensitivity of PC cells is regulated by autophagy and ROS pathway (8). Translocation of high-mobility group box 1 (HMGB1) was dependent on the generated ROS, meanwhile HMGB1 can induce autophagy (9).

High mobility group (HMG) proteins are a group of non-histone nuclear proteins (10). HMG proteins include three super-families named HMGB, HMGN, and HMGA (11). High-mobility group box 1 (HMGB1) is the most well-studied and abundant HMG protein. HMGB1 senses and coordinates the cellular stress response, which plays an important role not only inside of the cell as an autophagy sustainer, a DNA chaperone and protector from apoptotic cell death, but also outside the cell as a damage associated molecular pattern molecule (DAMP) (10). Human HMGB1 has 215 amino acid residues which forms two DNA binding domains (HMG A box, HMG B box) and a C-terminal acidic tail (12). All of these peculiarities make HMGB1 a critical molecular target in a variety of human diseases, such as cancer (13).

There are ~ 106 molecules of HMGB1 in each cell (14). The release of HMGB1 outside the cell membrane can be induced by cell stress or death. As a result, the HMGB1 in the extracellular fluid is called exogenous HMGB1 (15). Molecules released by dead or stressed cells can act as adjuvants or danger signals to the immune system. These signals are collectively known as damage-related molecular patterns (DAMPs) (16). Hallmark DAMPs have been extensively studied for its immunoadjuvant role in the spread of anti-tumor immune responses (17). HMGB1 has since been reevaluated to be an important DAMP protein (18). HMGB1 can be released into the extracellular matrix (ECM) by either granulocytes or necrotic cells to act as a cytokine/chemotaxis during cancer, infection, hypoxia, endotoxemia, and ischemia—reperfusion events (19). In addition to its nuclear and extracellular roles, cytoplasmic HMGB1 binds many proteins which are involved in cancer progression, autophagy, and is likely involved in the unconventional secretory pathway (20).

Several mechanisms have been proposed with regard to HMGB1 translocation from the cell nucleus to the cytoplasm and then subsequent release (21). Wnt signaling is the major pathway during embryonic development and continues throughout the life of an organism (22). Multifunctional serine/threonine kinase glycogen synthase kinase 3β (GSK-3β) is a crucial kinase for this process (23). GSK-3β is involved in multiple signaling pathways and in many processes of pathophysiology, such as differentiation, EMT, proliferation, metabolism, and inflammation (24). GSK-3β participates in a variety of human diseases, such as cancer (25). Some studies demonstrated that GSK-3β could affect EMT by regulating the Wnt pathway (26). However, the mechanism of GSK-3β action in EMT has not been fully clarified.

In this study, we investigated the role of exogenous HMGB1 in tumor proliferation and metastasis using human SW1990 and PANC-1 cells after radiotherapy. We explored the possible molecular mechanism, which could provide new strategies for the treatment of pancreatic cancer.

## Materials and Methods

### Materials

HMGB1 was purchased from Sigma (USA). The stock solution was aliquoted prior to storage to minimize freezing-thawing cycles. According to the instructions, we obtained the final concentration of 3.7 mg/mL HMGB1 mother liquor by adding 270.27 μL double distilled water into the original bottle with 1mg powder. Subsequently, we divided the mother liquor of HMGB1 into 10μL eppendorf tube and stored these in the refrigerator at −80°C. Before the experiment, a 10μL eppendorf tube of HMGB1 was diluted into 1μg/mL HMGB1 with PBS. During the experiment, 100ng/mL HMGB1 solution was finally obtained by dilution. Fetal bovine serum (FBS), DMEM/F12 was obtained from Gibco (USA). HMGB1 ELISA Kit was purchased from R&D systems (USA). Ethyl pyruvate (EP) was purchased from Sigma (USA). CFSE was purchased from BD (USA). Tris, glycine, TEMED, SDS, and acrylamide were obtained from Amresco (USA).

### Cell Culture

Pancreatic cancer cell lines (PANC-1 and SW1990) were purchased from the Chinese Academy of Sciences Shanghai Branch Cell Bank (Shanghai, China). PANC-1 and SW1990 cells were cultured in DMEM/F12 medium supplemented with 10% FBS, 5% penicillin, and 5% streptomycin at 37°C in a moist atmosphere with 5% CO_2_. When confluency of cells reached 70–80%, cells were detached and placed in additional flasks.

### Residual Cells Isolation

We collected residual PANC-1 cells 24 h after 1^*^4Gy radiation dose and residual SW1990 cells 24 h after 1^*^10Gy radiation dose. The living PANC-1 and SW1990 cells after radiotherapy were defined as residual pancreatic cancer cells. These residual pancreatic cancer cells were cultured in DMEM/F12 medium which was supplemented with 10% FBS and the cells grew as monolayers in a humidified atmosphere at 37°C, 5% CO_2_.

### Extraction of Supernatant After Radiation

The PANC-1 and SW1990 cells (2.5 × 10^5^ per well) were plated in 6-well plates and cultured in DMEM/F12 medium supplemented with 10% FBS, 5% penicillin, and 5% streptomycin at 37°C in a moist atmosphere with 5% CO_2_ for 24h after radiotherapy with a dose ranging from 0–12 Gy (0Gy, 4Gy, 8Gy, 10Gy, and 12Gy). Finally, we collected the supernatant of PANC-1 and SW1990 cells using sterile eppendorf tube.

### Enzyme Linked Immunosorbent Assay (ELISA)

The content of HMGB1 in the supernatant after radiotherapy was determined using HMGB1 ELISA kit. All steps were performed in a sterile environment. All reagents and sample dilution were prepared according to the instructions. Blank holes, standard holes and sample holes were set up, respectively. The supernatant of PANC-1 and SW1990 cells after radiotherapy with a dose ranging from 0–12 Gy were collected as samples. An amount of 100μl of standard or sample was added to each hole. All experiments were performed in triplicate and the data shown are representative.

### CFSE Staining

The cell suspensions were cultured in petri dish for 12 h to adherent state, and then the adherent PC cells were divided into three groups (control group, HMGB1 group, and HMGB1+ EP group). After collecting and washing via PBS three times, cells were incubated at 37°C in 5mM CFSE with fetal calf serum (FCS) free RPMI for 10 min. Labeling was stopped with FBS and cells were washed three times for further experiments. For the quantification of cell proliferation, cells were analyzed by flow cytometry. All experiments were performed in triplicate and the data shown are representative.

### Cell Migration Assay

We investigated the migration of PANC-1 and SW1990 cells by wound scratch assay. The PANC-1 and SW1990 cells (2.5 × 10^5^ per well) were plated in 6-well plates. A wound was scratched in the confluent cell layer using a 200 μL pipette tip after 24 h of incubation with PBS, 100 ng/ml HMGB1, and 100 ng/ml HMGB1+5μM EP (HMGB1 inhibitor). Cells were washed twice to remove detached cells and debris. Finally, the wound sizes were observed and measured after 0h, 12h, and 24h by EMS. All experiments were performed in triplicate and the data shown are representative.

### Western Blot Analysis

Cultured cancer cells were washed three times in phosphate-buffered saline (PBS) and lysed with RIPA buffer. Proteins were isolated by 10% sodium dodecyl sulfate–polyacrylamide gel (SDS-PAGE). Subsequently, proteins were transferred to nitrocellulose membranes and incubated with primary antibodies all night at 4°C followed by secondary antibodies for 1 h at room temperature. Blots were visualized using an ECL detection system (Amersham, Piscataway, NJ, USA). LANE-1D Analyzer (Sage BJ, China) was used to quantify the intensity of protein bands. The relative intensity of β-actin, total GSK3β, and Histone 4 was calculated by normalization. Histone 4 was used as a loading control. All experiments were performed in triplicate and the data shown are representative.

### Nude Mice

The animal study was reviewed and approved by the Committee on the Use of Live Animals for Affiliated Hospital of Nanjing University of Chinese Medicine (No. 2019NL-161-02). All male nude mice were fostered in animal experiment center of Affiliated Hospital of Nanjing University of Chinese Medicine in compliance with the Guideline for the Care and Use of Laboratory Animals, where the condition is held at the constant temperature (25–27°C), constant humidity (45–50%), fresh air, and without special pathogenic bacteria. We established the pancreatic tumor model of nude mice by injecting SW1990 cells (2 × 10^6^ per mouse) into the flanks of the mice subcutaneously. when the tumor reached about 5 mm in diameter, all subcutaneous tumors of nude mice were treated by radiotherapy (3^*^10Gy). After radiotherapy for 6 days, nine nude mice were divided into three groups and then received hypodermic injection of physiological saline, 100 ng/ml HMGB1, and 100 ng/ml HMGB1+ 5mmol/L EP around subcutaneous tumor. The tumors were measured every 3 days. After 2 weeks of treatment, all nude mice were sacrificed, and the tumors were collected for further analysis. All experiments were performed in triplicate and the data shown are representative.

### Statistical Analysis

All statistical analyses were performed using GraphPad Prism 5 (GraphPad Software Inc. San Diego, CA, USA). Data were presented as the mean ± standard deviation (SD). Two-tailed unpaired *t* test was carried out to compare the mean of two independent groups. Among three or more groups, one-way analysis of variance (ANOVA) was used. A *p* < 0.05 indicated a statistically significant difference.

## Results

### HMGB1 Is Overexpressed in the Supernatant of PC Cells After Radiotherapy

**Figure 1:**
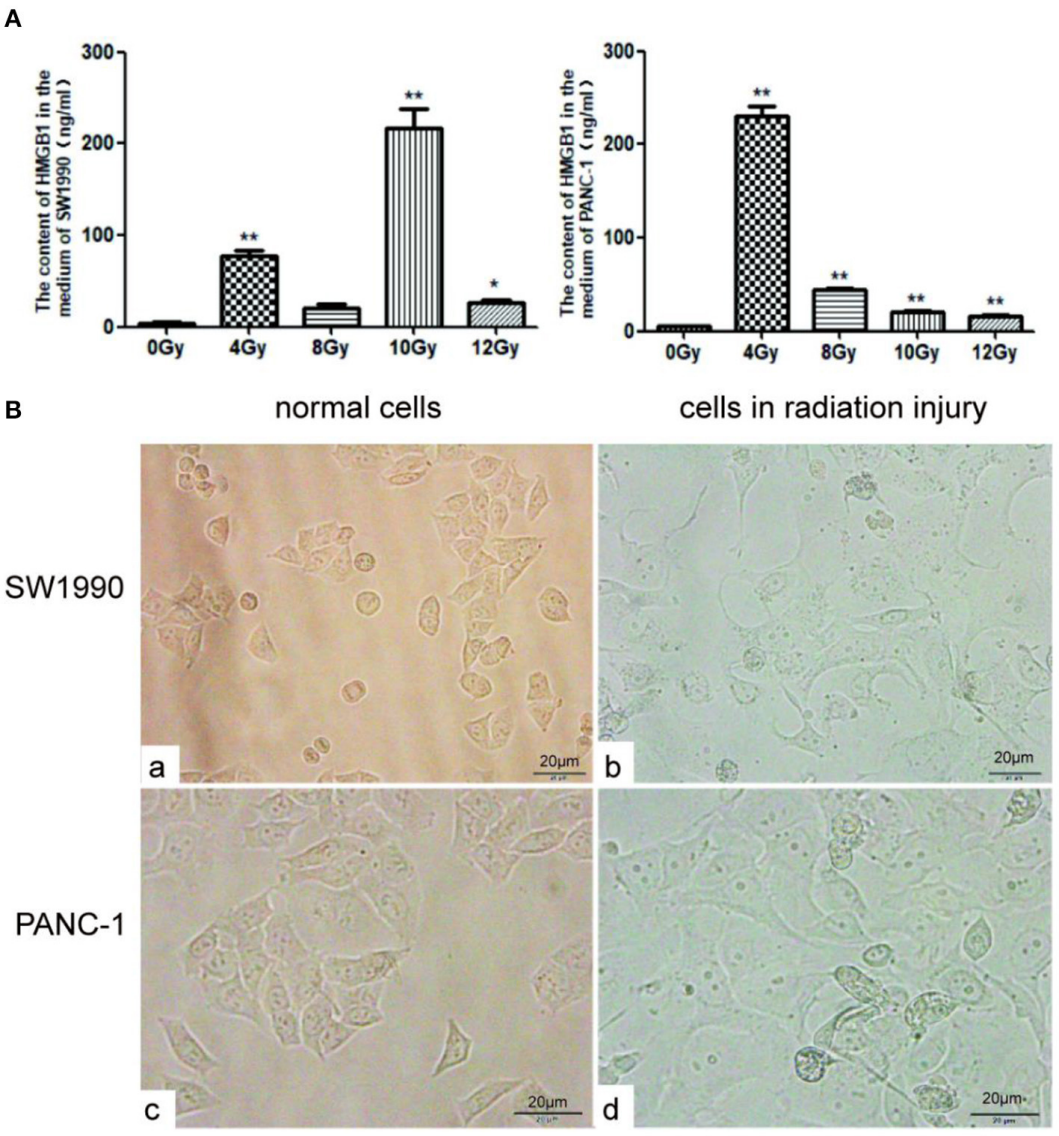
HMGB1 is overexpressed in the supernatant of PC cells after radiotherapy. **(A)** The content of HMGB1 in the supernatant was compared between control group and radiotherapy groups (0, 4, 8, 10 or 12Gy) using ELISA assay. Gy is the unit of radiation dose. **(B)** The images of normal SW1990 cells (a), SW1990 cells after radiation injury (b), normal PANC-1 cells (c), and PANC-1 cells after radiation injury (d) were collected by electron microscope. *p < *0.05*, **p < *0.01* vs. control group.

After treatment with different radiation doses (0, 4, 8, 10, or 12Gy), the concentrations of HMGB1 in the culture medium of PC cell lines (PANC-1 and SW1990) were measured using ELISA. As shown in Figure 1A, the concentration of HMGB1 in the culture medium of PC cells after radiotherapy was much higher than control group (0Gy). As shown in Figure 1A, the concentration of HMGB1 [(217.3 ± 35.0) ng/ml] in the culture medium of SW1990 cells reached the maximum when radiation dose was 10 Gy; meanwhile, the concentration of HMGB1 [(229.9 ± 18.3) ng/ml] in the culture medium of PANC-1 cells reached the maximum when radiation dose was 4 Gy. Therefore, we concluded that the optimal radiation doses for SW1990 and PANC-1 cells were 10Gy and 4Gy, respectively (Figure 1A).

Under normal culture conditions, the shape of SW1990 and Pan-1 cells were fusiform (Figure 1Ba,b). However, after cultured in the supernatant with an optimal radiation dose for 24h, SW1990 and Pan-1 cells in radiation injury grew faster and larger than normal group (Figure 1Bc,d).

### Exogenous HMGB1 Promotes the Proliferation of PC Cells

**Figure 2:**
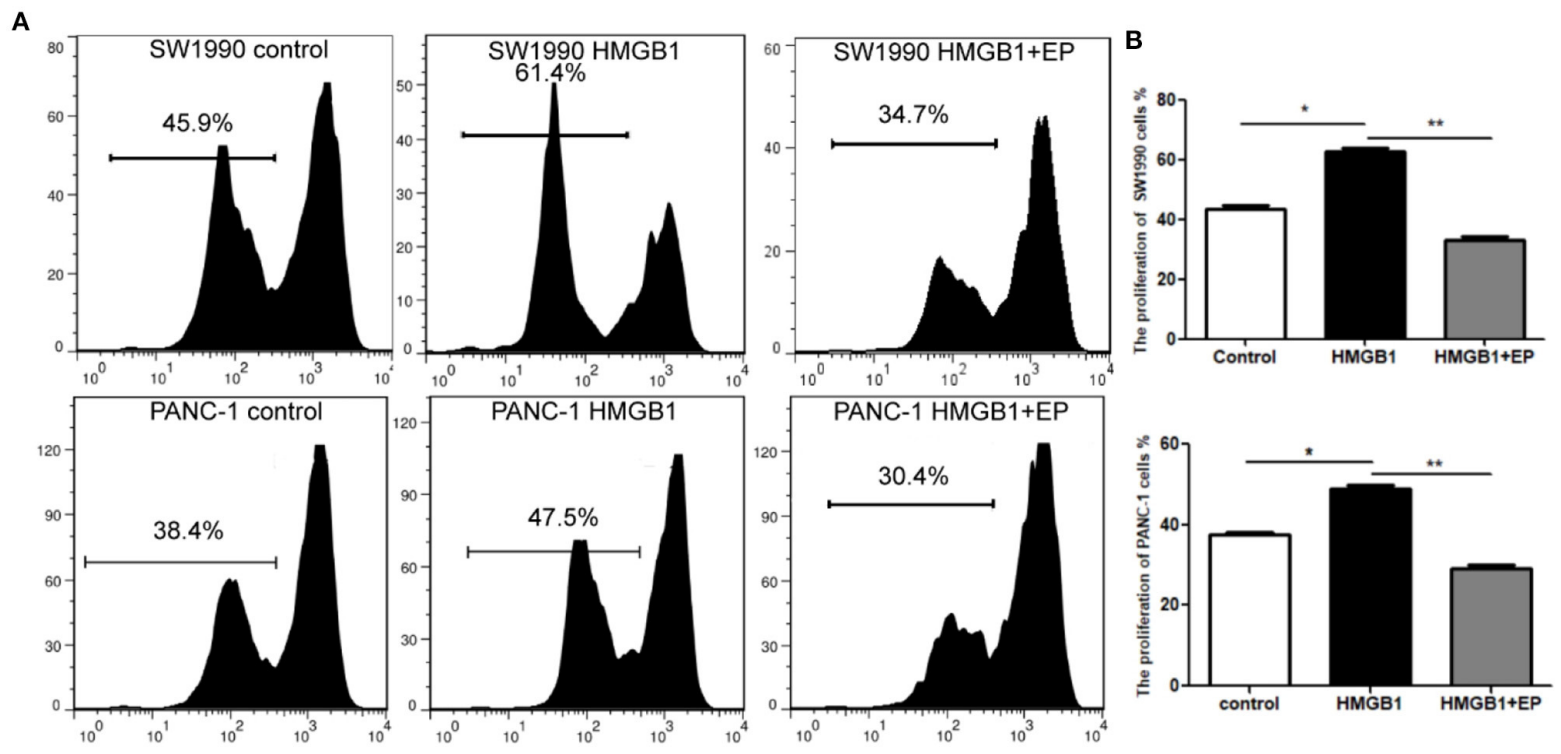
Impact of exogenous HMGB1 on the proliferation of PC cells. **(A)** Cell proliferation was compared between control group, HMGB1 group and HMGB1+EP group by flow cytometry. **(B)** Data of proliferation rates are statistically analyzed between control group, HMGB1 group and HMGB1+EP group. *p < *0.05*, **p < *0.01* vs. control group.

SW1990 and PANC-1 cells were collected and cultured in different medium (PBS, 100ng/ml HMGB1 and 100ng/ml HMGB1+5μM EP). Cells were collected after 96h and detected using CFSE staining. Our results showed that the proliferation rate of the HMGB1 group is significantly higher than the control group, while the proliferation rate of the EP (HMGB1 inhibitor) group was down-regulated (Figure 2). We thereby speculate that exogenous HMGB1 plays a critical role in the proliferation of SW1990 and Pan-1 cells in radiation injury.

### Exogenous HMGB1 Promotes the Migration of PC Cells

**Figure 3:**
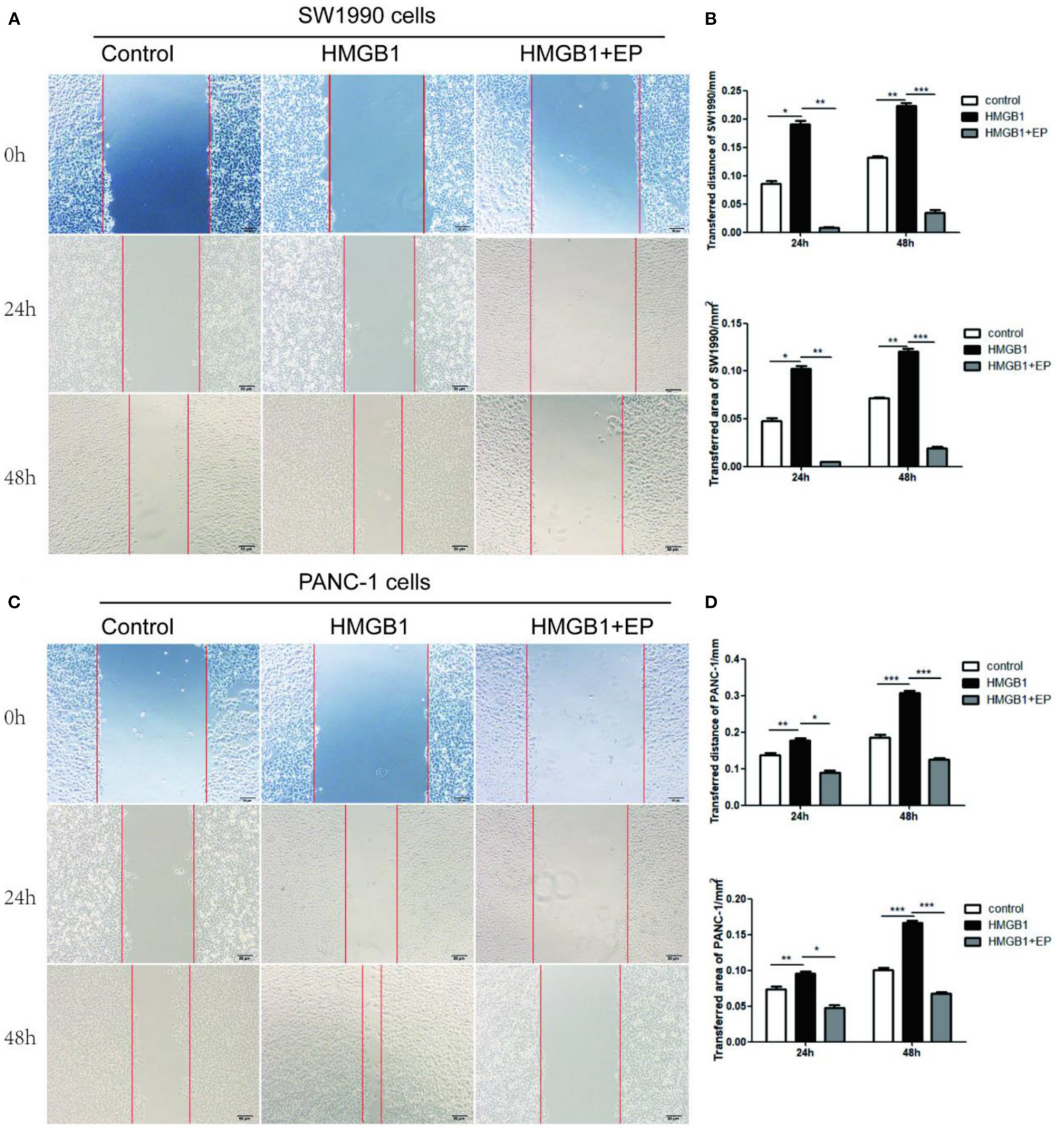
Exogenous HMGB1 promote the migration of PC cells. **(A,B)** The migration of SW1990 cells was executed by using wound healing. The ability of migration was compared among control group, HMGB1 group, HMGB1+EP group for 0,24,48h. **(C,D)** The migration of PANC-1 cells was determined by using wound healing. The migrative ability was compared among control group, HMGB1 group, HMGB1+EP group for 0,24,48h. *p < *0.05*, **p < *0.01*, ***p < *0.001* vs. control group.

The effect of exogenous HMGB1 on the migration of PC cells was evaluated using wound healing assay. As shown in Figures 3A,C, the migration distance of HMGB1 group (100ng/ml HMGB1) in SW1990 and PANC-1 cells was larger than control group (PBS) and HMGB1+EP group (100ng/ml HMGB1+5μM EP) after 24 and 48h of culture. Interestingly, the migrative ability of HMGB1 + EP group was significantly decreased as compared to HMGB1 group (Figures 3B,D, *p*<*0.05*).

### Exogenous HMGB1 Affects the EMT of PC Cells

**Figure 4:**
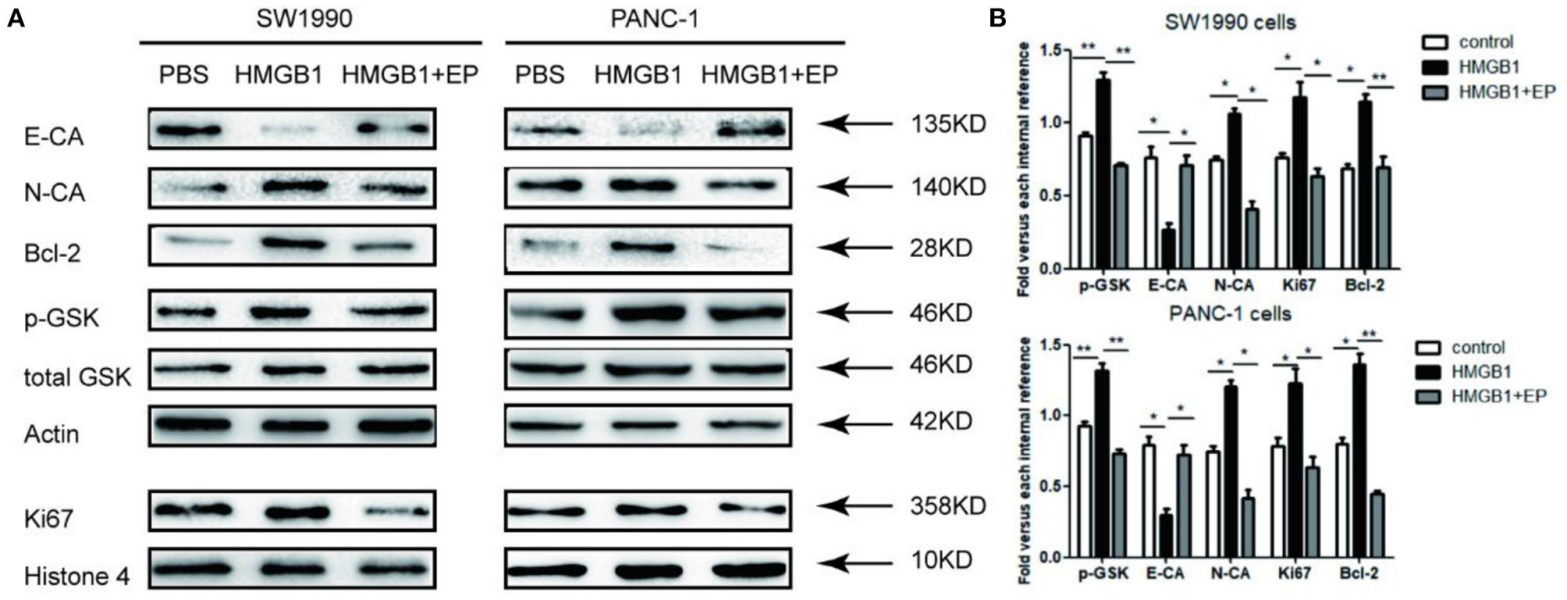
Impact of exogenous HMGB1 on EMT and Wnt pathway of pancreatic cancer cells. **(A)** Western blots for E-CA, N-CA, Bcl-2, p-GSK 3β and Ki67 levels in control group, HMGB1 group or HMGB1+EP group. Actin, total GSK, and Histone 4 were used as internal control. **(B)** Data are presented as the mean ± SD, *n* = 3. *p < *0.05*, **p < *0.01* vs. control group.

As shown in Figure 4, after treatment with 100ng/ml exogenous HMGB1, the western blot results showed the activation of p-GSK 3β and the up-regulation of N-CA, Bcl-2, and Ki67 in response to HMGB1 stimulation, while E-CA expression was down-regulated in pancreatic cancer cells in response to HMGB1 stimulation. Additionally, HMGB1+EP group (100ng/ml HMGB1+5μM EP) showed the opposite results.

### Exogenous HMGB1 Promotes the Proliferation of Tumor Postradiotherapy *in vivo*

**Figure 5:**
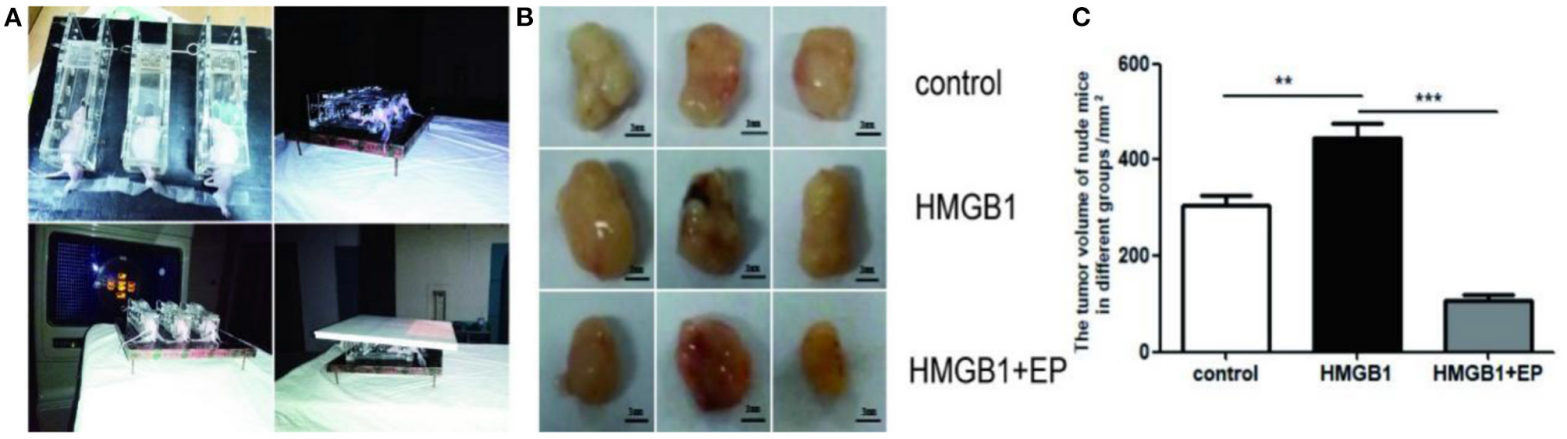
Exogenous HMGB1 promotes residual tumor proliferation *in vivo*. **(A)** Nude mice fixed and used local radiotherapy. **(B,C)** The tumor volumes of nude mice in different group (control group, HMGB1 group, HMGB1+EP group). **P < *0.01*, ***P < *0.001* vs. control group.

An amount of 2 × 10^6^ SW1990 cells were injected into the flanks of the mice subcutaneously. when the tumor reached about 5 mm in diameter, all subcutaneous tumors of nude mice were treated by radiotherapy (3^*^10Gy). The influence of exogenous HMGB1 on the proliferation of residual tumor was observed. As shown in Figure 5A, tumor-bearing nude mice received local radiotherapy. As shown in Figure 5B, exogenous HMGB1 treatment significantly promoted the proliferation of tumors after radiotherapy as compared to the control group. However, the proliferation of tumors was significantly inhibited in HMGB1+EP group compared to the control group (Figure 5C). Overall, the analysis of tumor volume in different groups showed that exogenous HMGB1 promotes residual tumor proliferation *in vivo*.

## Discussion

**Figure 6:**
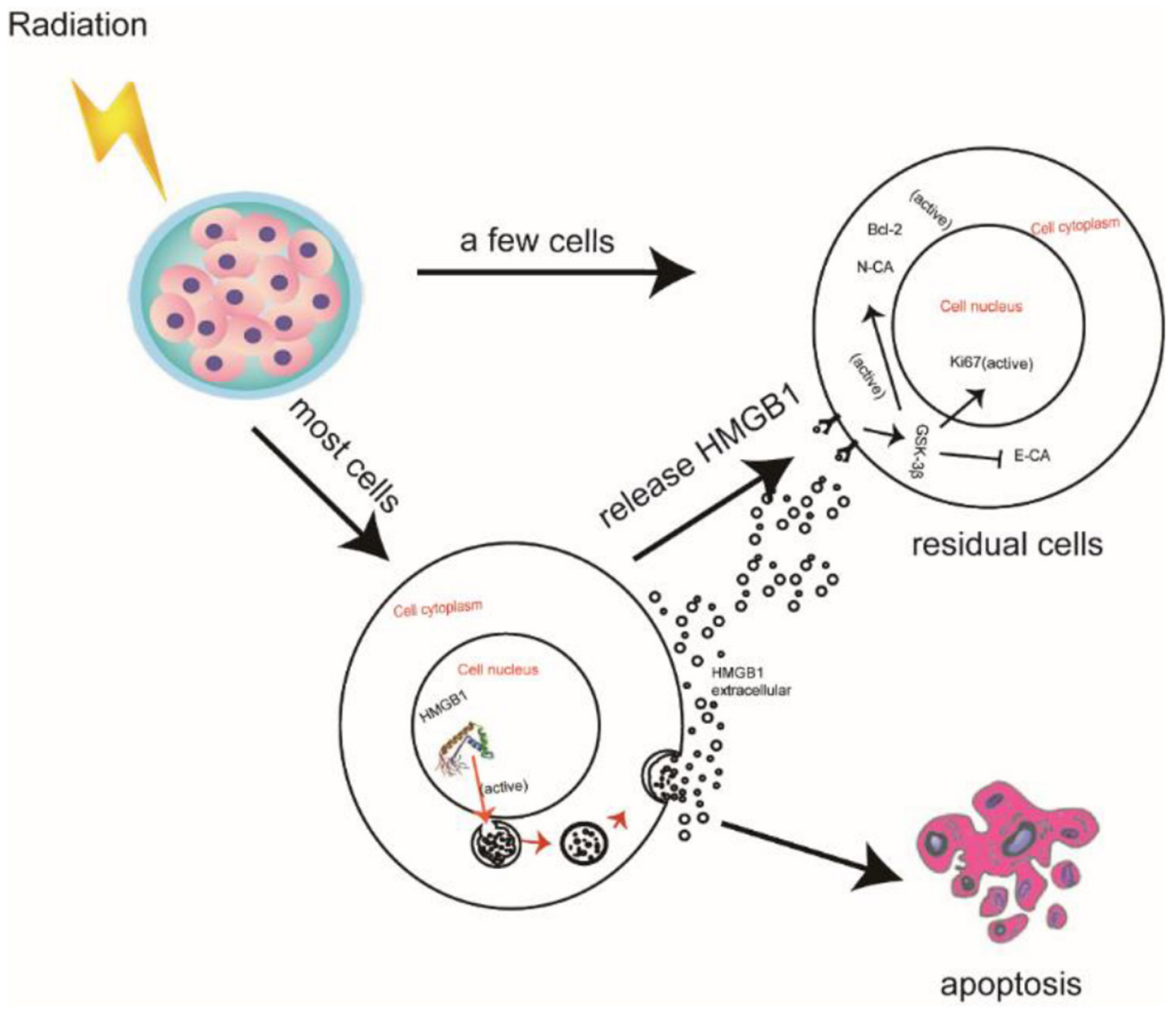
Schematic illustration of HMGB1 promoting the proliferation and migration of residual pancreatic cancer cells after radiotherapy.

The prognosis of PC is very poor due to its insidious onset, deep anatomic position, challenge in early diagnosis, inoperability, and poor response to radio-chemotherapy (27). With an estimated 232,000 new cases per year, PC is one of the most common malignancies all around the world (28). Unfortunately, the cure rate for these “curable” PC patients is only about 20% (29). The role of radiotherapy for treatment of PC has been discussed for many years and has been deemed extremely unsatisfactory (30). Radiotherapy causes apoptosis in most PC cells, but a few of PC cells survive. This can be attributed to the radio-resistance of residual PC cells. Additionally, the apoptotic cells release HMGB1 into culture medium after radiotherapy. In this study, we investigated the potential value of exogenous HMGB1 after radiotherapy which we believe contributes to the proliferation and migration of residual PC cells by autocrine regulation based on our study results (Figure 6).

HMGB1 is not only a chromatin-associated nuclear protein, but also an extracellular damage-associated molecular-pattern molecule. HMGB1 is overexpressed in tumor cells and triggers inflammation, tumor migration and cell migration (31). Some studies have shown that HMGB1 plays a pivotal role by RAGE or the toll-like receptors TLR2 and TLR4, and the activation of these receptors can result in the activation of NF-kB. These activated signals then enhance the productions of angiogenic factors and proinflammatory cytokines in hematopoietic and endothelial cells (32, 33). PC cells had the highest release of HMGB1 as compared to different cancer cell lines (e.g., breast, lung and bladder) (34). HMGB1 plays a pivotal role in the progression and development of pancreatic cancer.

Epithelial-mesenchymal transition (EMT) is an early event in tumor growth and especially dissemination (35). EMT is associated with tumor growth and especially dissemination. EMT has been found in many malignant tumors including liver, pancreatic, gastric, colorectal, and breast cancer (36, 37). Cells expressing EMT not only acquire the ability to migrate, but also undergo a host of changes in vital cell cycle check-point inhibitors/promotors (38). In human pancreatic tumor samples, the expression of E-cadherin is decreased, with a corresponding increase in N-cadherin expression (39). E-cadherin is regarded as a tumor metastasis suppressor gene as well as a key gene in the EMT process (40). In our study, the expression of E-cadherin was down-regulated with subsequent upregulation of N-cadherin in the HMGB1 group as compared to control (*p* < 0.05).

Wnt signaling is the major pathway of embryogenesis and cancer development, while GSK-3 is a crucial kinase for this process (41, 42). Glycogen synthase kinase (GSK-3β) plays a pivotal role in Wnt signaling pathway by the phosphorylation procedure and p-GSK 3β regulates the downstream target genes (43). It is reported that HMGB1 undergoes spontaneous EMT through the GSK-3β signaling pathway (44). In this study, we found that p-GSK 3β was activated in the HMGB1 group as compared to the HMGB1+EP group.

By a series of experiments, we speculate that radiation induces the activation of HMGB1 in PC cells. After hydroxylation of HMGB1 activated, HMGB1 is released extracellularly *via* vesicles. Radiation injured cells release a plethora of HMGB1 extracellularly. Residual PC cells have many receptors on the cytomembrane, ultimately leading to GSK-3β phosphorylation.

There were several limitations. First, we only investigated the role of exogenous HMGB1 in tumor proliferation and metastasis using PC cells. We also did not measure the percentage of the PANC-1 and SW1990 cells that survived after different doses of radiation therapy. We will investigate whether the doses corresponded with cell survival in the near future. In addition, immunotherapy for cancer has made remarkable progress in recent years (45, 46); we will investigate the potential value of immunotherapies in the development and prevention of PC in the near future.

In conclusion, our data suggest that exogenous HMGB1 promotes the proliferation and metastasis of pancreatic cancer cells after radiotherapy. The HMGB1 in residual PC cells may induce the activation of EMT by increasing p-GSK 3β activity and activating the Wnt signal path. In future experiments, we will identify more HMGB1 target proteins. We hope to further elucidate the various molecular mechanisms. Additionally, we will select more PC cell lines in follow-up experiments. Ultimately, we hope to provide a new treatment strategy for PC that recurs after radiotherapy.

## Data Availability Statement

The original contributions presented in the study are included in the article/Supplementary Material, further inquiries can be directed to the corresponding author/s.

## Ethics Statement

The animal study was reviewed and approved by the Committee on the Use of Live Animals for Affiliated Hospital of Nanjing University of Chinese Medicine.

## Author Contributions

All authors listed have made a substantial, direct and intellectual contribution to the work, and approved it for publication.

## Funding

This study was funded by National Natural Science Foundation of China (Grant Nos. 82171925, 81771899, and 81471705) and the Administration of Traditional Chinese Medicine of Jiangsu Province (No. ZD201907).

## Conflict of Interest

The authors declare that the research was conducted in the absence of any commercial or financial relationships that could be construed as a potential conflict of interest.

## Publisher's Note

All claims expressed in this article are solely those of the authors and do not necessarily represent those of their affiliated organizations, or those of the publisher, the editors and the reviewers. Any product that may be evaluated in this article, or claim that may be made by its manufacturer, is not guaranteed or endorsed by the publisher.
