# Supplementary figures and images for: Exogenous HMGB1 Promotes the Proliferation and Metastasis of Pancreatic Cancer Cells
Source: Front Med (Lausanne). 2021 Nov 3;8:756988. doi: 10.3389/fmed.2021.756988 (PMC8595098; doi:10.3389/fmed.2021.756988)

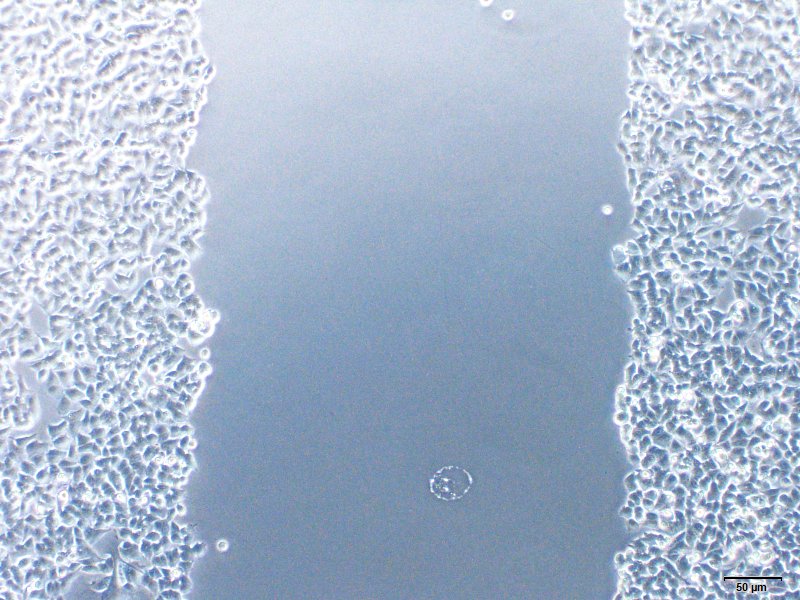

Supplement: Supplementary file 1 [file Data_Sheet_1.ZIP › Original files/original gels/cell microscopy image/Figure 3 cell/Panc-1 HMGB1 0h.jpg]

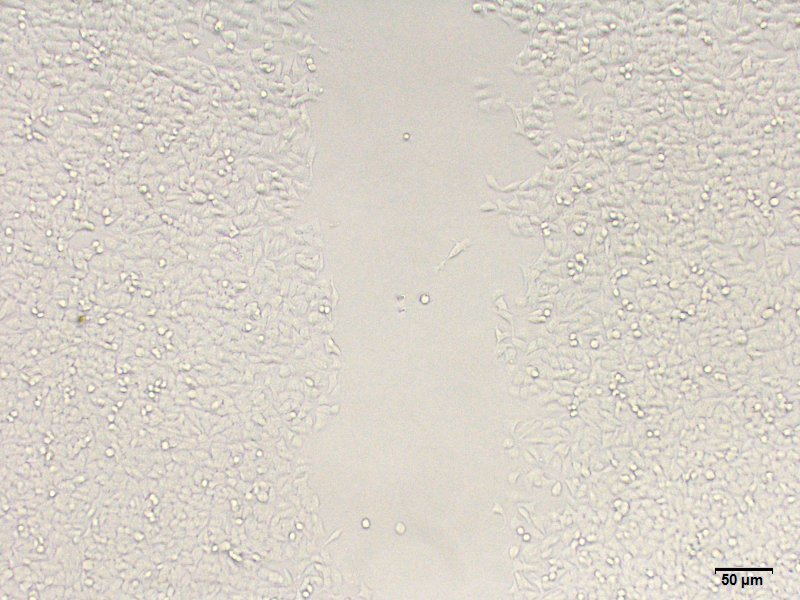

Supplement: Supplementary file 1 [file Data_Sheet_1.ZIP › Original files/original gels/cell microscopy image/Figure 3 cell/Panc-1 HMGB1 24h.jpg]

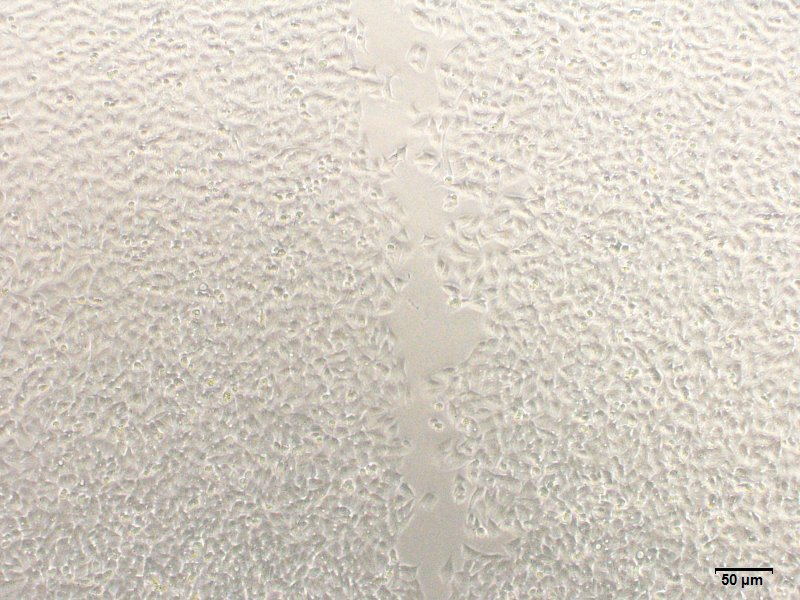

Supplement: Supplementary file 1 [file Data_Sheet_1.ZIP › Original files/original gels/cell microscopy image/Figure 3 cell/Panc-1 HMGB1 48h.jpg]

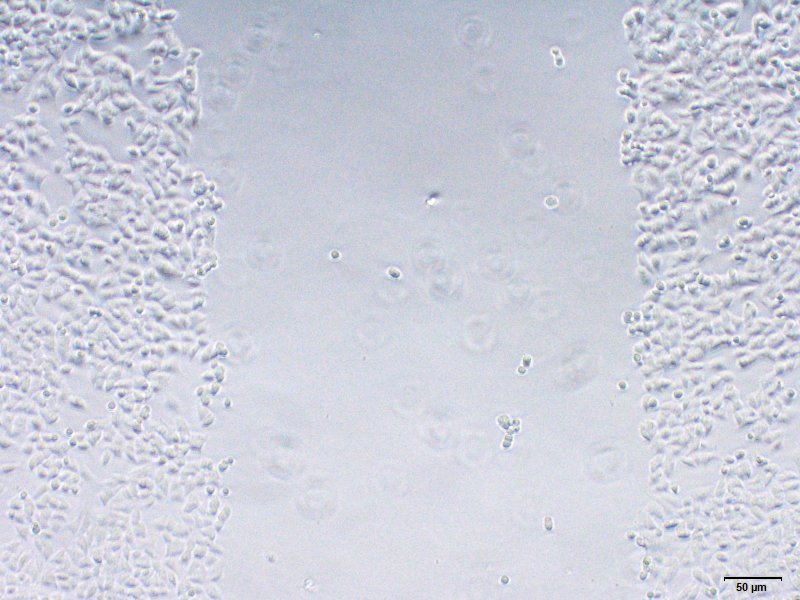

Supplement: Supplementary file 1 [file Data_Sheet_1.ZIP › Original files/original gels/cell microscopy image/Figure 3 cell/Panc-1 HMGB1+EP 0h.jpg]

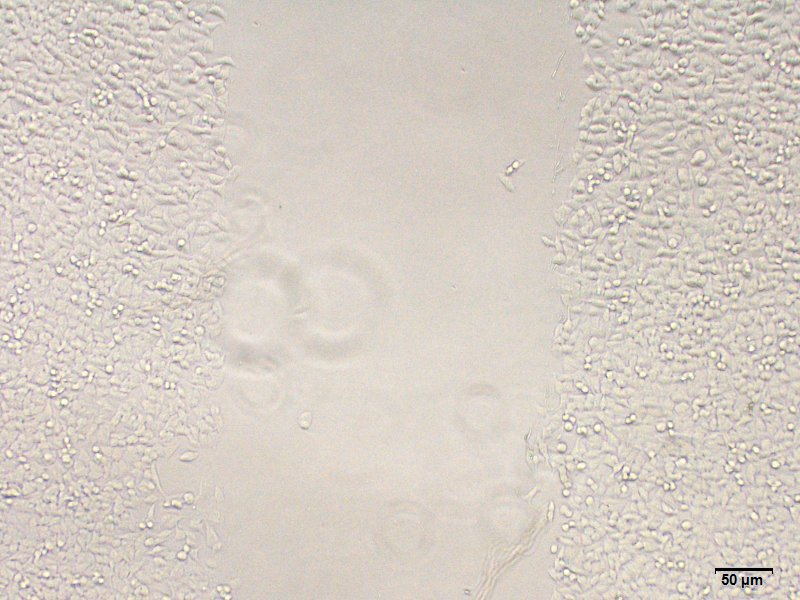

Supplement: Supplementary file 1 [file Data_Sheet_1.ZIP › Original files/original gels/cell microscopy image/Figure 3 cell/Panc-1 HMGB1+EP 24h.jpg]

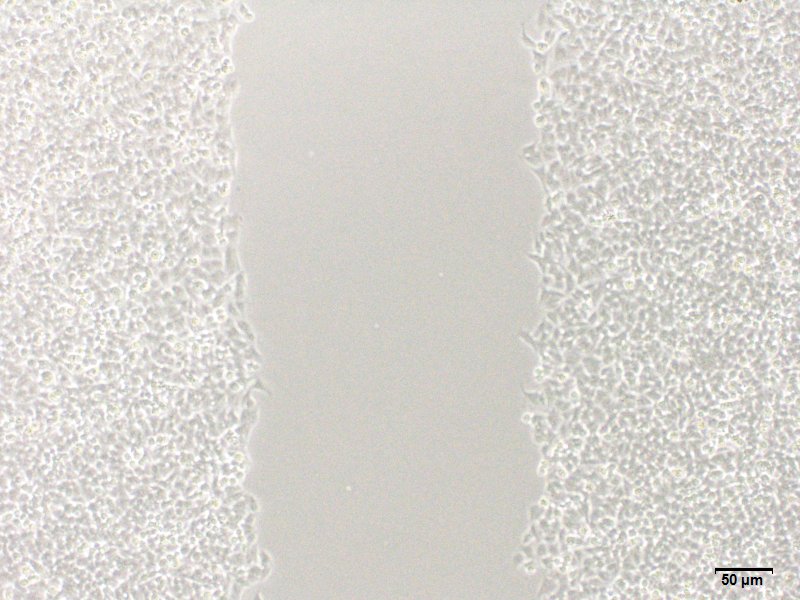

Supplement: Supplementary file 1 [file Data_Sheet_1.ZIP › Original files/original gels/cell microscopy image/Figure 3 cell/Panc-1 HMGB1+EP 48h.jpg]

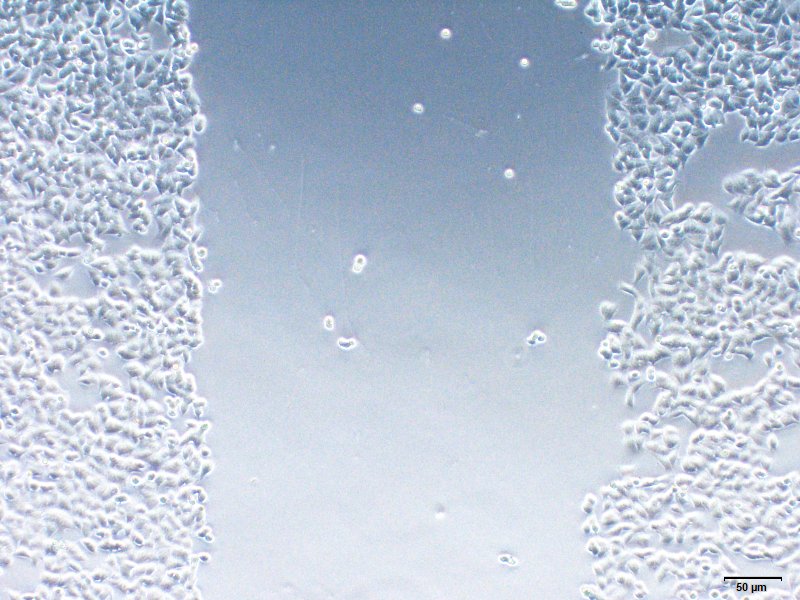

Supplement: Supplementary file 1 [file Data_Sheet_1.ZIP › Original files/original gels/cell microscopy image/Figure 3 cell/Panc-1 control 0h.jpg]

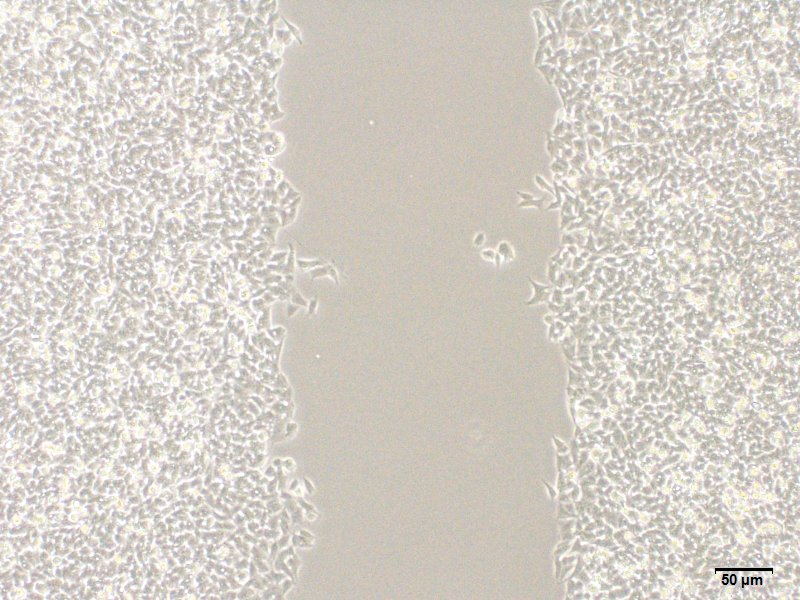

Supplement: Supplementary file 1 [file Data_Sheet_1.ZIP › Original files/original gels/cell microscopy image/Figure 3 cell/Panc-1 control 24h.jpg]

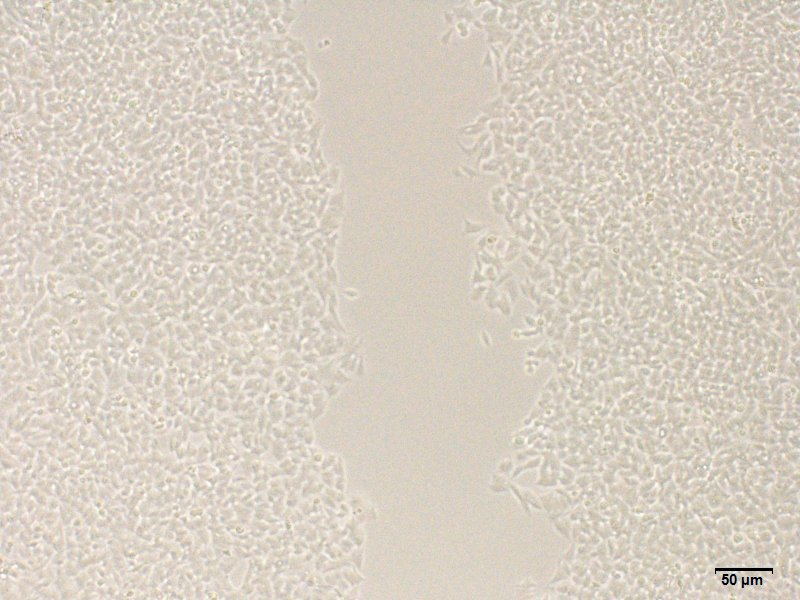

Supplement: Supplementary file 1 [file Data_Sheet_1.ZIP › Original files/original gels/cell microscopy image/Figure 3 cell/Panc-1 control 48h.jpg]

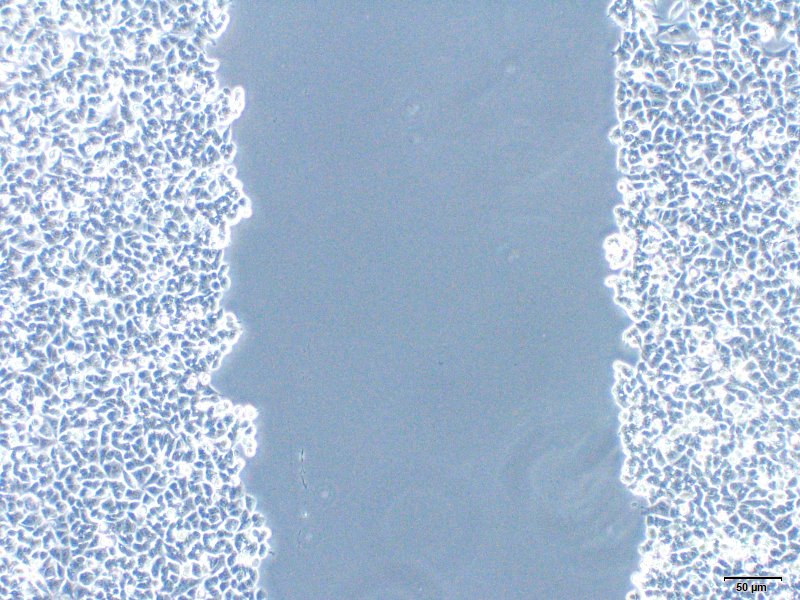

Supplement: Supplementary file 1 [file Data_Sheet_1.ZIP › Original files/original gels/cell microscopy image/Figure 3 cell/SW1990 HMGB1 0h.jpg]

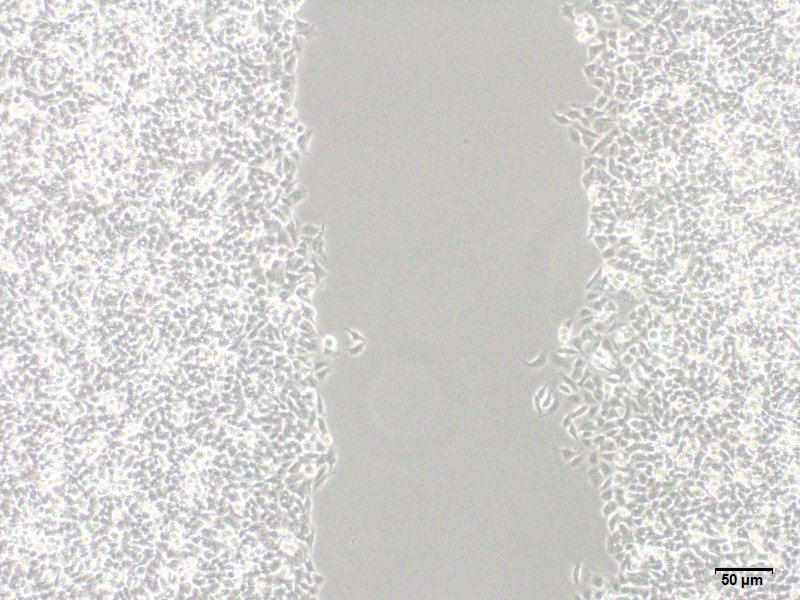

Supplement: Supplementary file 1 [file Data_Sheet_1.ZIP › Original files/original gels/cell microscopy image/Figure 3 cell/SW1990 HMGB1 24h.jpg]

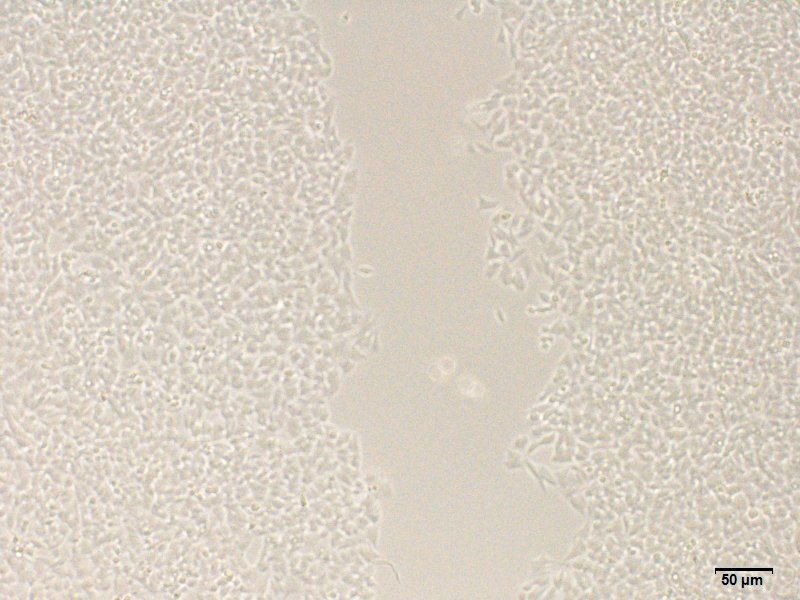

Supplement: Supplementary file 1 [file Data_Sheet_1.ZIP › Original files/original gels/cell microscopy image/Figure 3 cell/SW1990 HMGB1 48h.jpg]

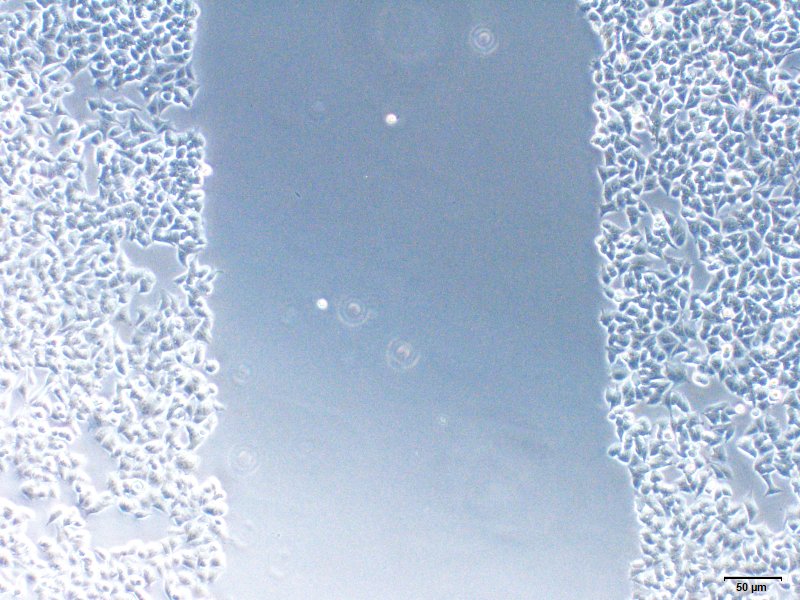

Supplement: Supplementary file 1 [file Data_Sheet_1.ZIP › Original files/original gels/cell microscopy image/Figure 3 cell/SW1990 HMGB1+EP 0h.jpg]

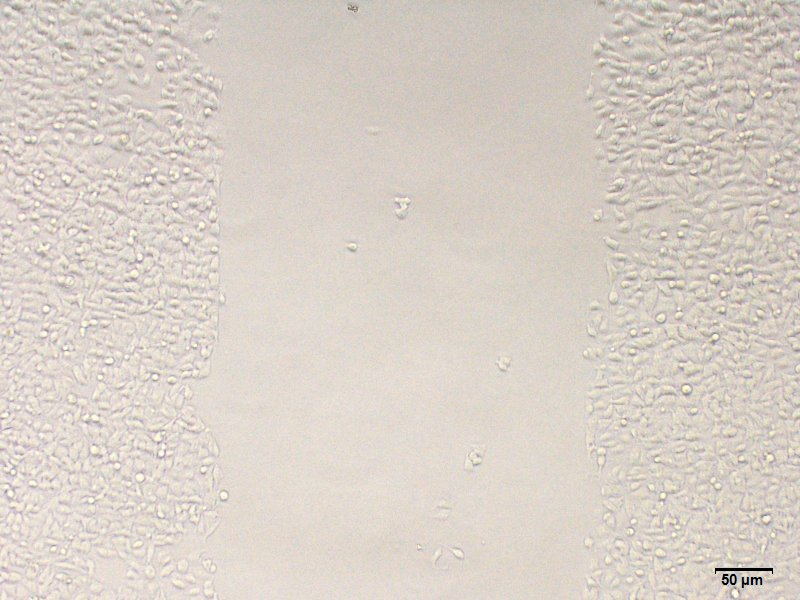

Supplement: Supplementary file 1 [file Data_Sheet_1.ZIP › Original files/original gels/cell microscopy image/Figure 3 cell/SW1990 HMGB1+EP 24h.jpg]

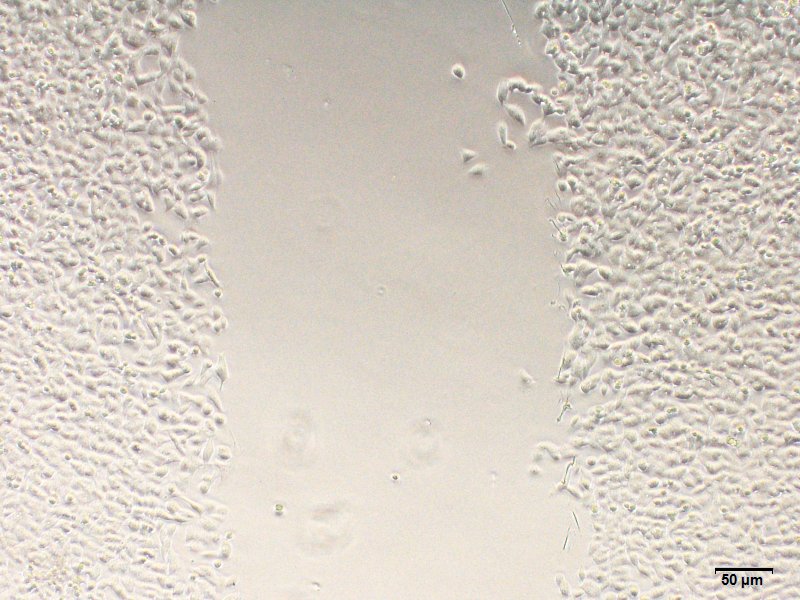

Supplement: Supplementary file 1 [file Data_Sheet_1.ZIP › Original files/original gels/cell microscopy image/Figure 3 cell/SW1990 HMGB1+EP 48h.jpg]

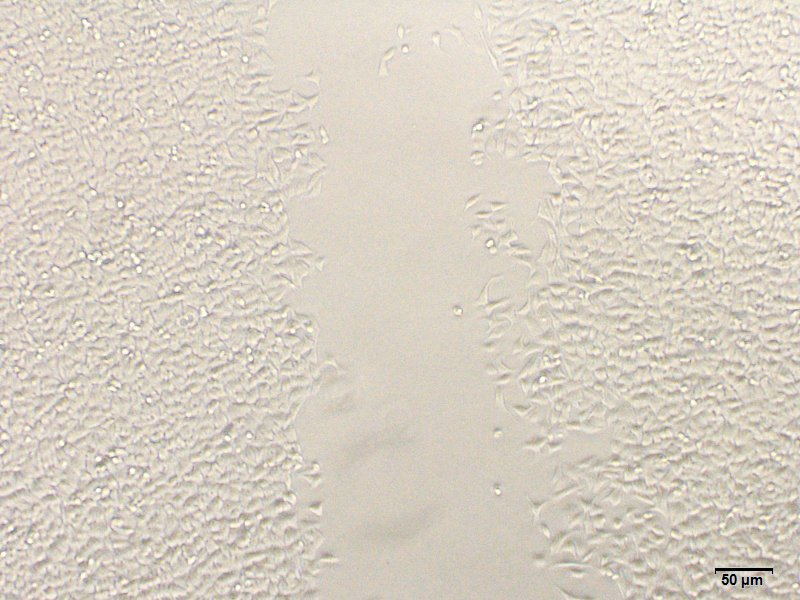

Supplement: Supplementary file 1 [file Data_Sheet_1.ZIP › Original files/original gels/cell microscopy image/Figure 3 cell/SW1990 control 48h.jpg]

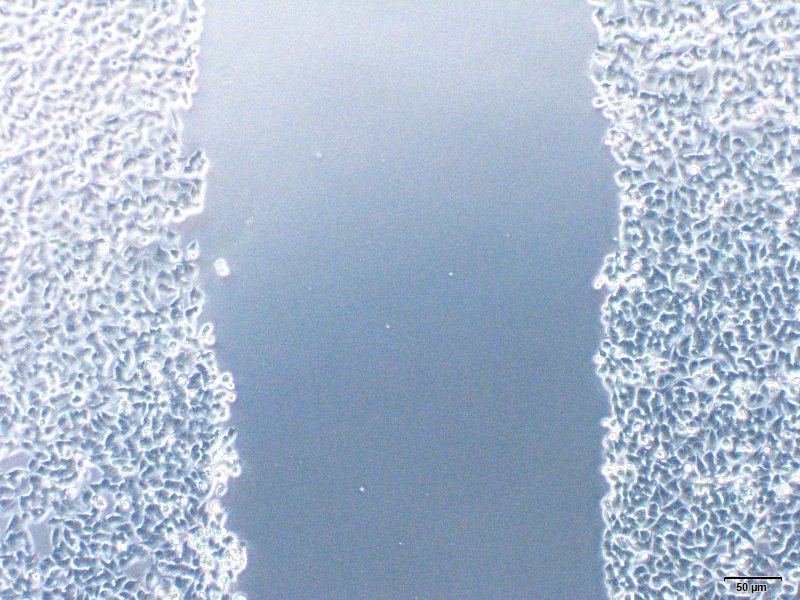

Supplement: Supplementary file 1 [file Data_Sheet_1.ZIP › Original files/original gels/cell microscopy image/Figure 3 cell/SW1990 control 0h.jpg]

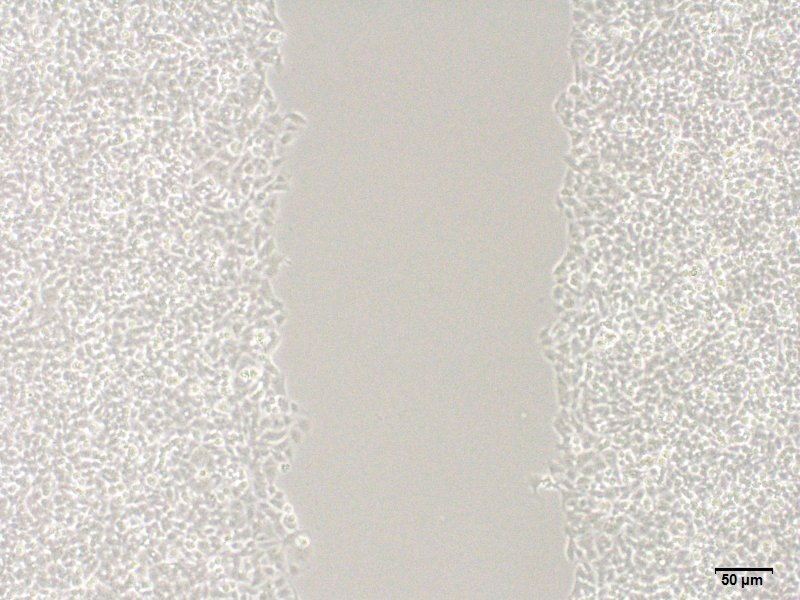

Supplement: Supplementary file 1 [file Data_Sheet_1.ZIP › Original files/original gels/cell microscopy image/Figure 3 cell/SW1990 control 24h.jpg]

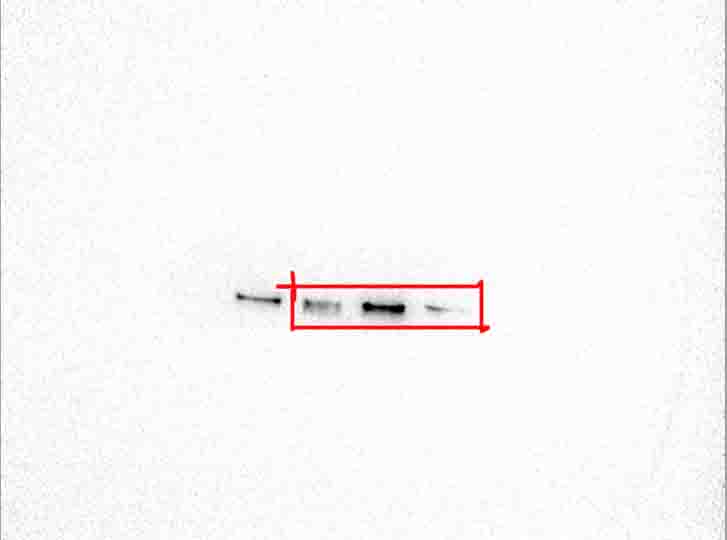

Supplement: Supplementary file 1 [file Data_Sheet_1.ZIP › Original files/original gels/original gels/Panc-1 Bcl-2 big picture required.jpg]

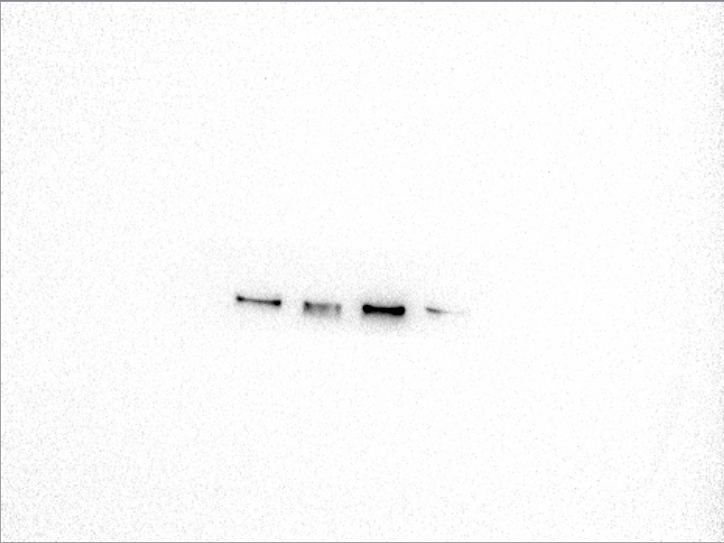

Supplement: Supplementary file 1 [file Data_Sheet_1.ZIP › Original files/original gels/original gels/Panc-1 Bcl-2 big picture.jpg]

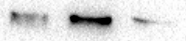

Supplement: Supplementary file 1 [file Data_Sheet_1.ZIP › Original files/original gels/original gels/Panc-1 Bcl-2.jpg]

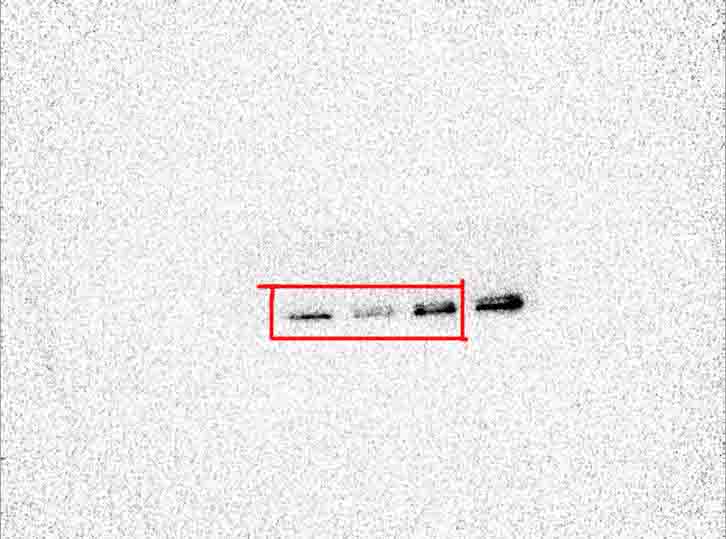

Supplement: Supplementary file 1 [file Data_Sheet_1.ZIP › Original files/original gels/original gels/Panc-1 E-CA big picture - required.jpg]

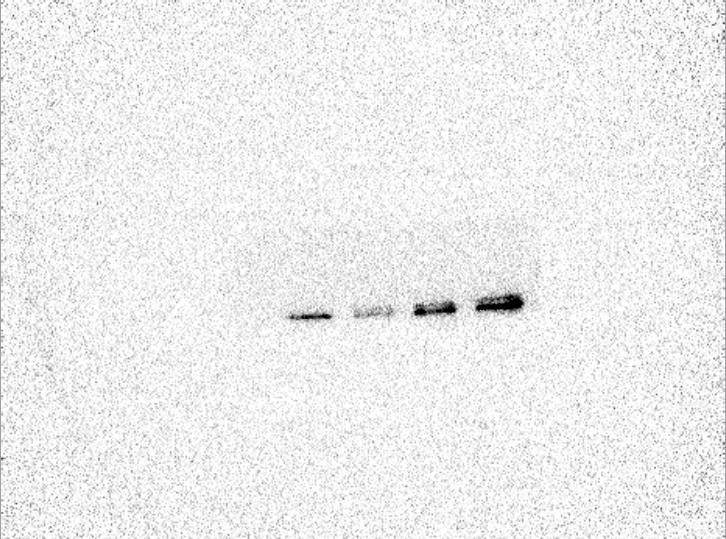

Supplement: Supplementary file 1 [file Data_Sheet_1.ZIP › Original files/original gels/original gels/Panc-1 E-CA big picture.jpg]

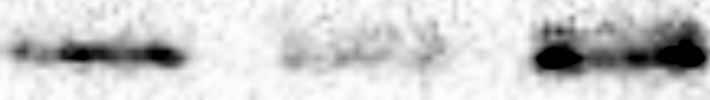

Supplement: Supplementary file 1 [file Data_Sheet_1.ZIP › Original files/original gels/original gels/Panc-1 E-CA.jpg]

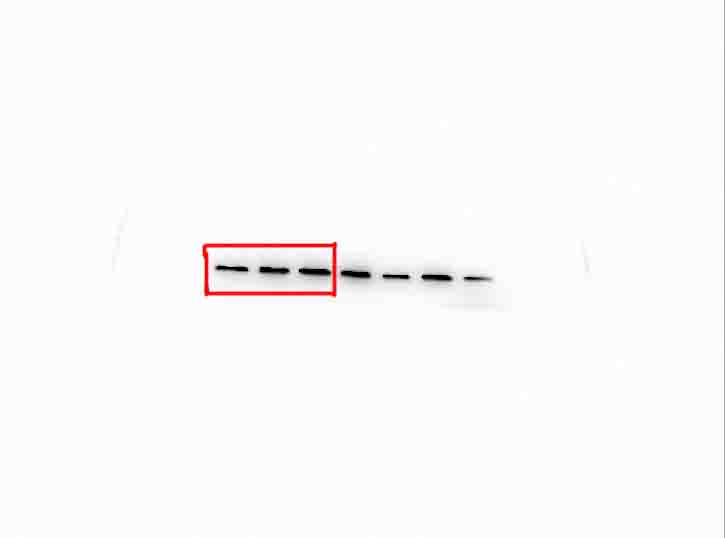

Supplement: Supplementary file 1 [file Data_Sheet_1.ZIP › Original files/original gels/original gels/Panc-1 Hoston 4 big picture - required.jpg]

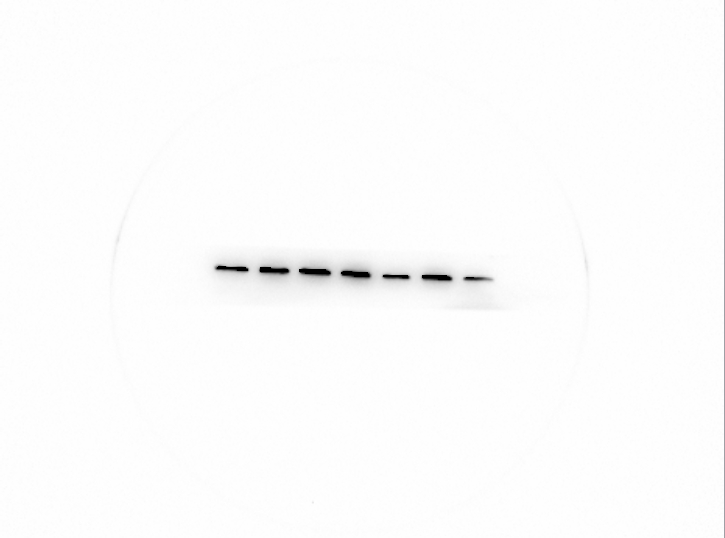

Supplement: Supplementary file 1 [file Data_Sheet_1.ZIP › Original files/original gels/original gels/Panc-1 Hoston 4 big picture.jpg]

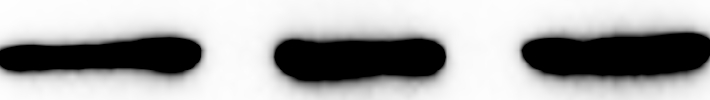

Supplement: Supplementary file 1 [file Data_Sheet_1.ZIP › Original files/original gels/original gels/Panc-1 Hoston 4.jpg]

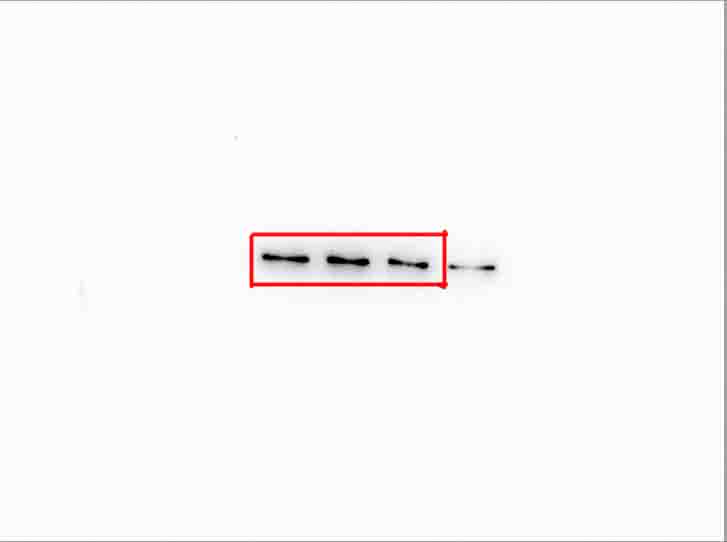

Supplement: Supplementary file 1 [file Data_Sheet_1.ZIP › Original files/original gels/original gels/Panc-1 Ki67 big picture - required.jpg]

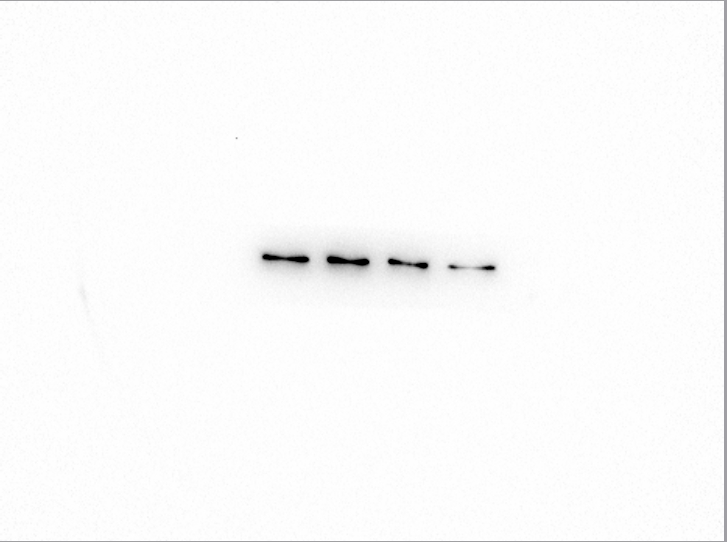

Supplement: Supplementary file 1 [file Data_Sheet_1.ZIP › Original files/original gels/original gels/Panc-1 Ki67 big picture.jpg]

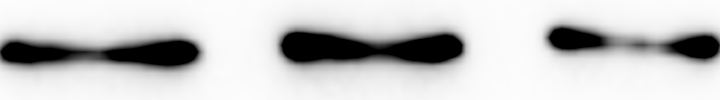

Supplement: Supplementary file 1 [file Data_Sheet_1.ZIP › Original files/original gels/original gels/Panc-1 Ki67.jpg]

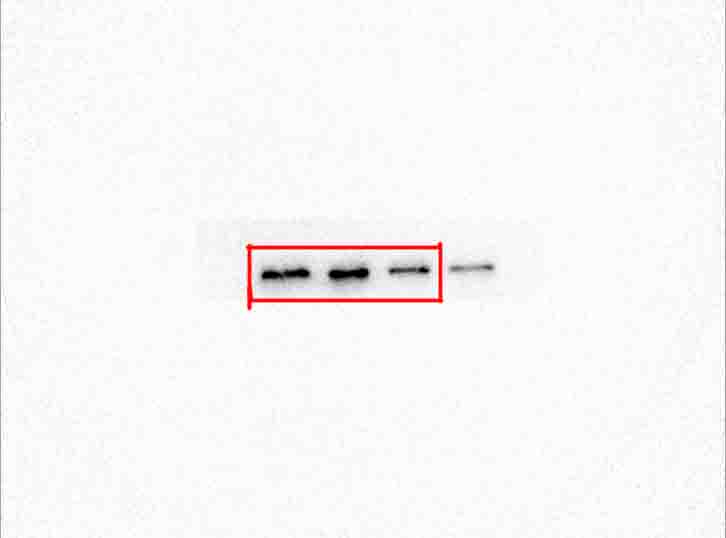

Supplement: Supplementary file 1 [file Data_Sheet_1.ZIP › Original files/original gels/original gels/Panc-1 N-CA big picture - required.jpg]

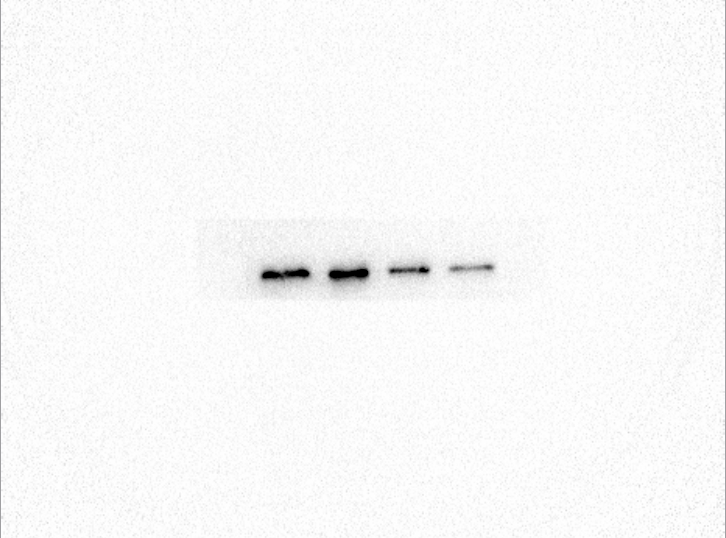

Supplement: Supplementary file 1 [file Data_Sheet_1.ZIP › Original files/original gels/original gels/Panc-1 N-CA big picture.jpg]

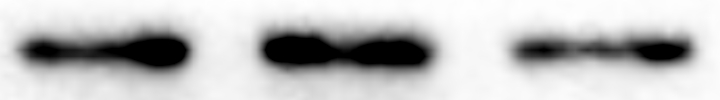

Supplement: Supplementary file 1 [file Data_Sheet_1.ZIP › Original files/original gels/original gels/Panc-1 N-CA.jpg]

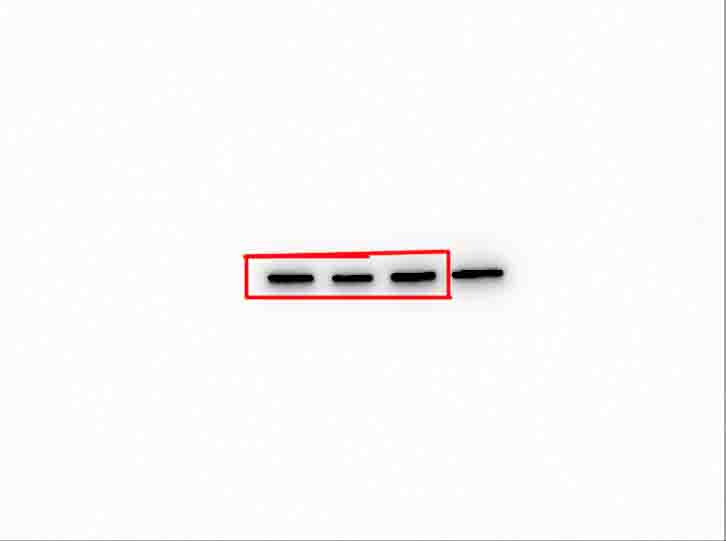

Supplement: Supplementary file 1 [file Data_Sheet_1.ZIP › Original files/original gels/original gels/Panc-1 actin big picture-required.jpg]

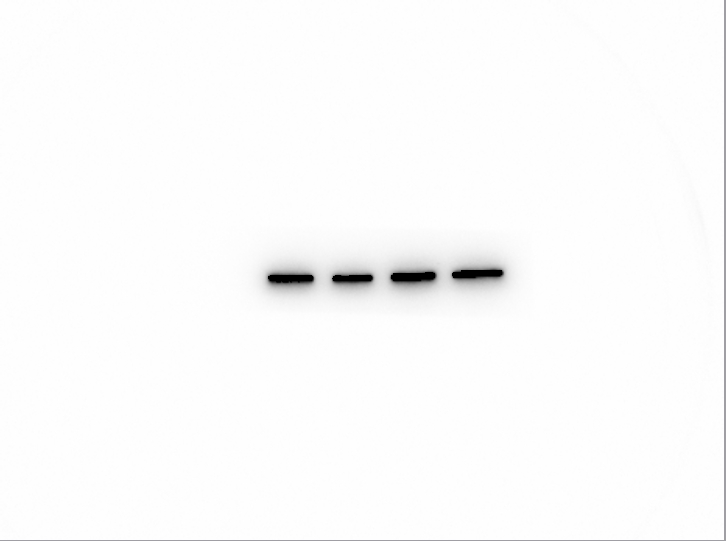

Supplement: Supplementary file 1 [file Data_Sheet_1.ZIP › Original files/original gels/original gels/Panc-1 actin big picture.jpg]

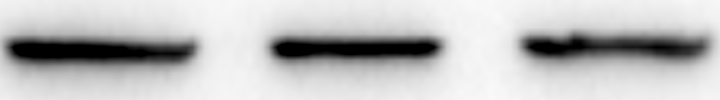

Supplement: Supplementary file 1 [file Data_Sheet_1.ZIP › Original files/original gels/original gels/Panc-1 actin.jpg]

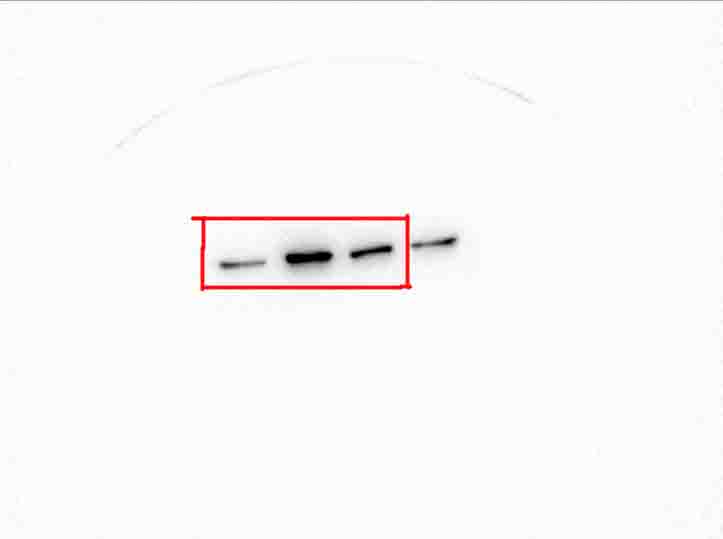

Supplement: Supplementary file 1 [file Data_Sheet_1.ZIP › Original files/original gels/original gels/Panc-1 p-GSK big picture - required.jpg]

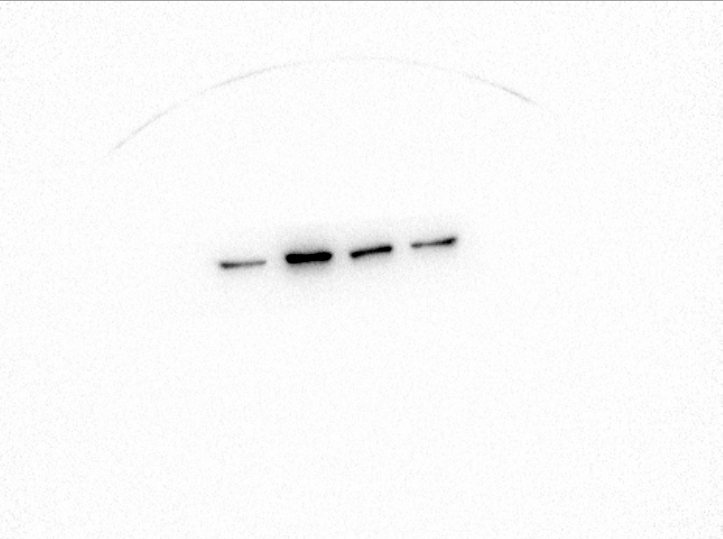

Supplement: Supplementary file 1 [file Data_Sheet_1.ZIP › Original files/original gels/original gels/Panc-1 p-GSK big picture.jpg]

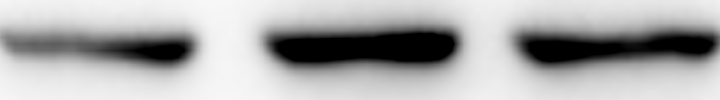

Supplement: Supplementary file 1 [file Data_Sheet_1.ZIP › Original files/original gels/original gels/Panc-1 p-GSK.jpg]

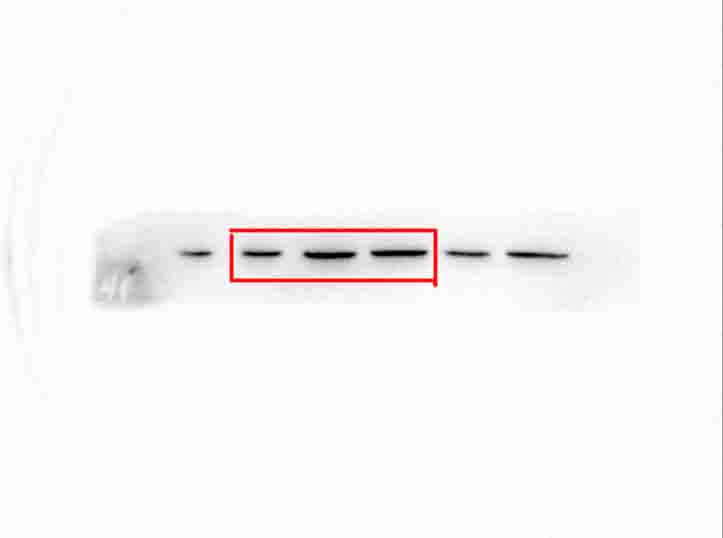

Supplement: Supplementary file 1 [file Data_Sheet_1.ZIP › Original files/original gels/original gels/Panc-1 total GSK big picture - required.jpg]

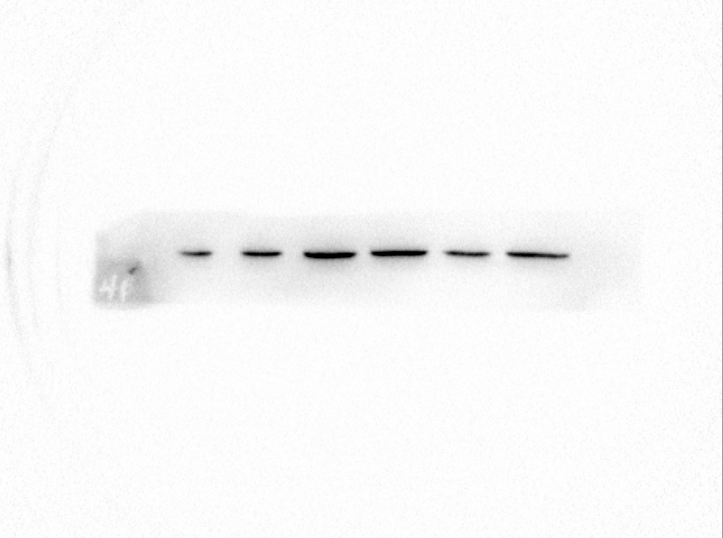

Supplement: Supplementary file 1 [file Data_Sheet_1.ZIP › Original files/original gels/original gels/Panc-1 total GSK big picture.jpg]

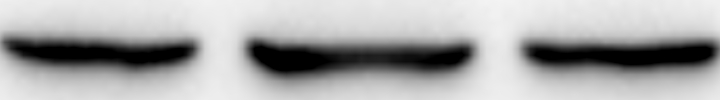

Supplement: Supplementary file 1 [file Data_Sheet_1.ZIP › Original files/original gels/original gels/Panc-1 total GSK.jpg]

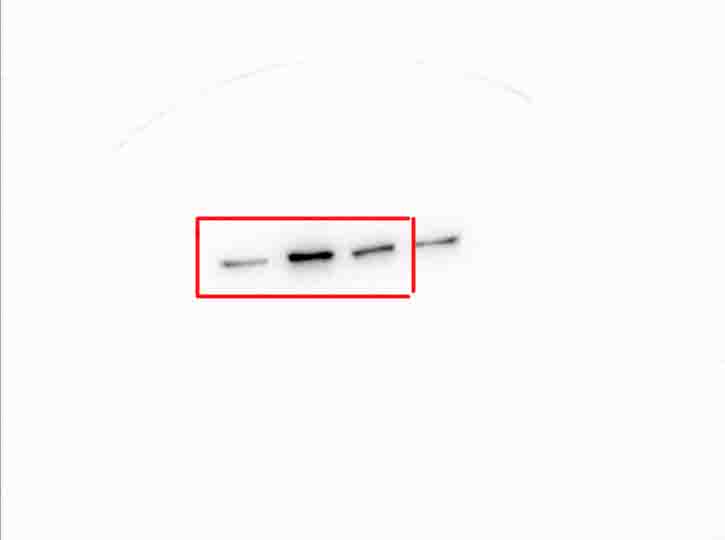

Supplement: Supplementary file 1 [file Data_Sheet_1.ZIP › Original files/original gels/original gels/SW1990 Bcl-2 big piture required.jpg]

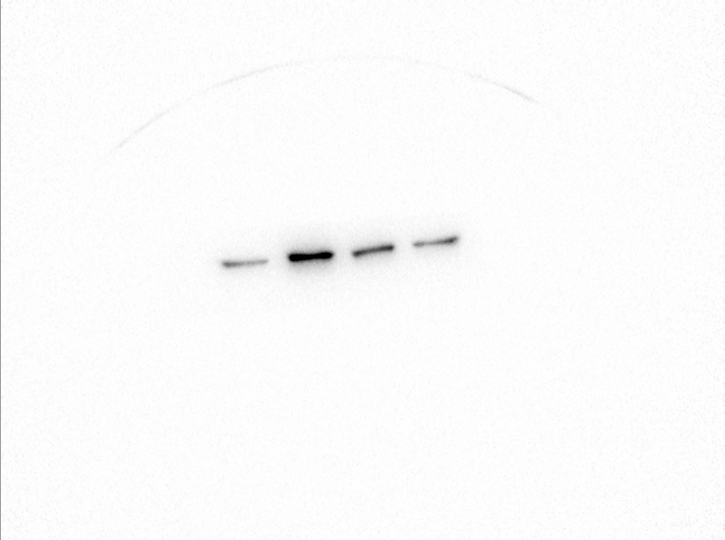

Supplement: Supplementary file 1 [file Data_Sheet_1.ZIP › Original files/original gels/original gels/SW1990 Bcl-2 big piture.jpg]

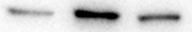

Supplement: Supplementary file 1 [file Data_Sheet_1.ZIP › Original files/original gels/original gels/SW1990 Bcl-2.jpg]

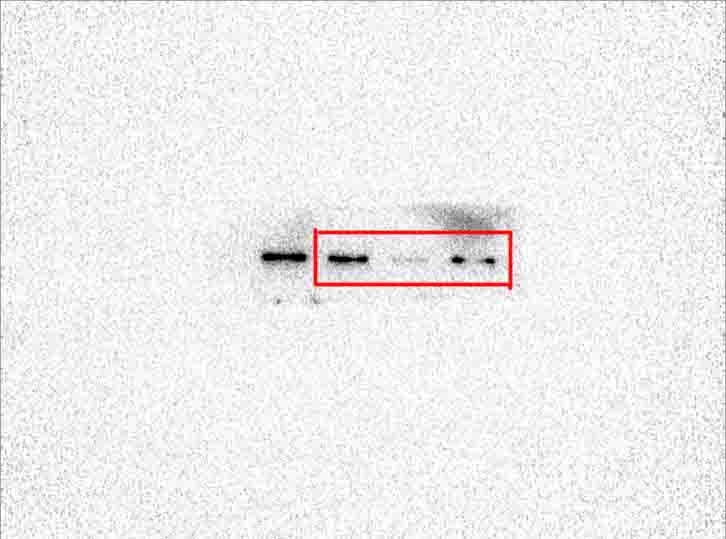

Supplement: Supplementary file 1 [file Data_Sheet_1.ZIP › Original files/original gels/original gels/SW1990 E-CA big picture - required.jpg]

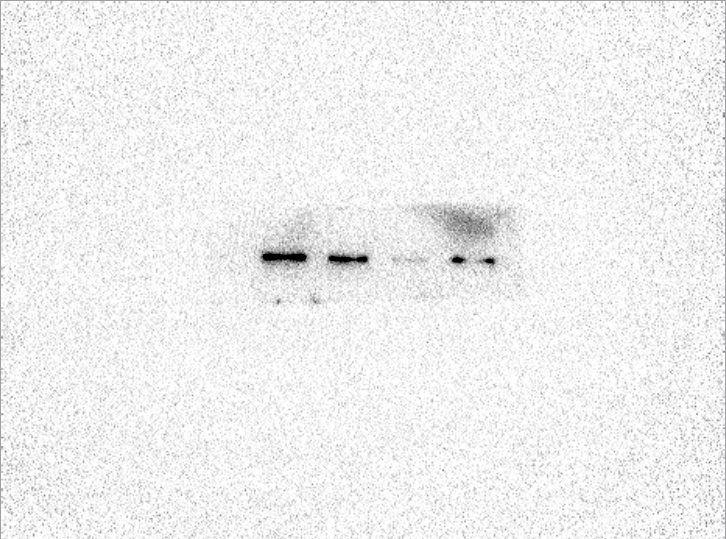

Supplement: Supplementary file 1 [file Data_Sheet_1.ZIP › Original files/original gels/original gels/SW1990 E-CA big picture.jpg]

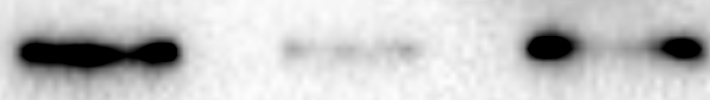

Supplement: Supplementary file 1 [file Data_Sheet_1.ZIP › Original files/original gels/original gels/SW1990 E-CA.jpg]

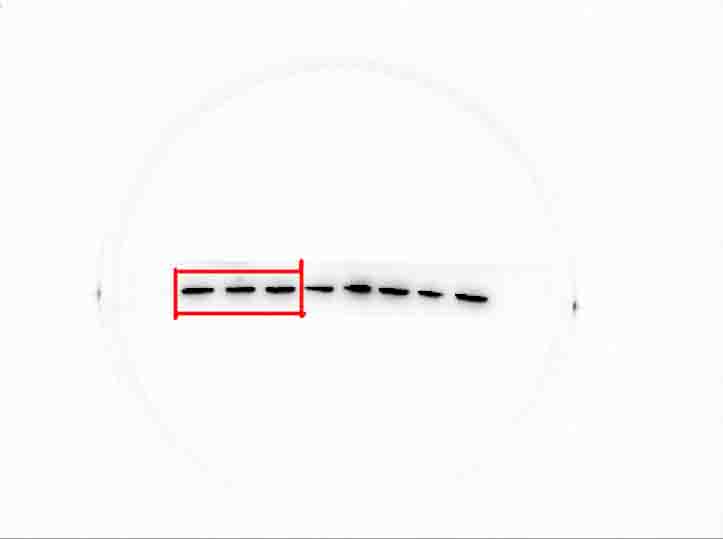

Supplement: Supplementary file 1 [file Data_Sheet_1.ZIP › Original files/original gels/original gels/SW1990 Hostone 4 big picture - required.jpg]

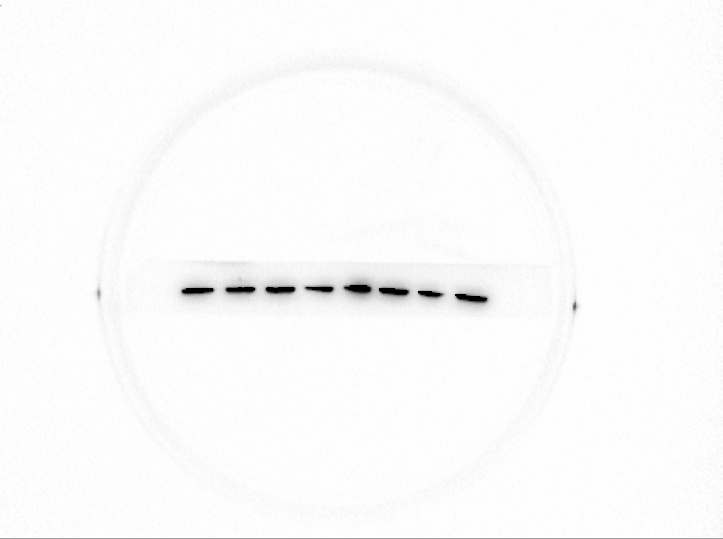

Supplement: Supplementary file 1 [file Data_Sheet_1.ZIP › Original files/original gels/original gels/SW1990 Hostone 4 big picture.jpg]

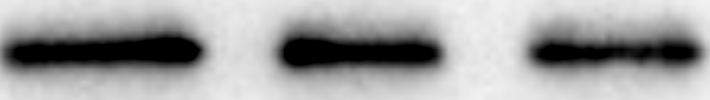

Supplement: Supplementary file 1 [file Data_Sheet_1.ZIP › Original files/original gels/original gels/SW1990 Hostone 4.jpg]

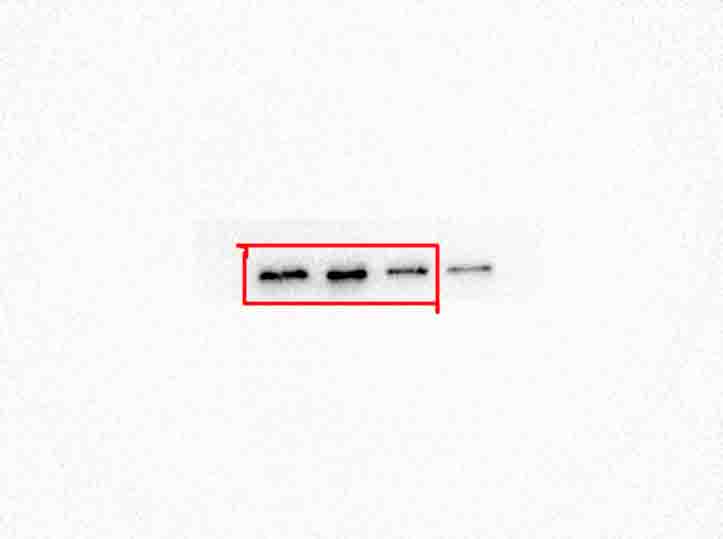

Supplement: Supplementary file 1 [file Data_Sheet_1.ZIP › Original files/original gels/original gels/SW1990 Ki67 big picture - required.jpg]

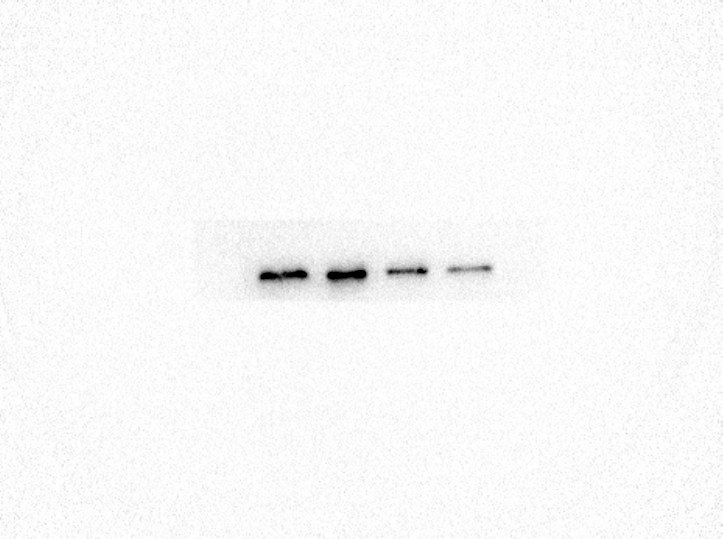

Supplement: Supplementary file 1 [file Data_Sheet_1.ZIP › Original files/original gels/original gels/SW1990 Ki67 big picture.jpg]

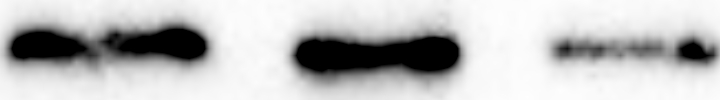

Supplement: Supplementary file 1 [file Data_Sheet_1.ZIP › Original files/original gels/original gels/SW1990 Ki67.jpg]

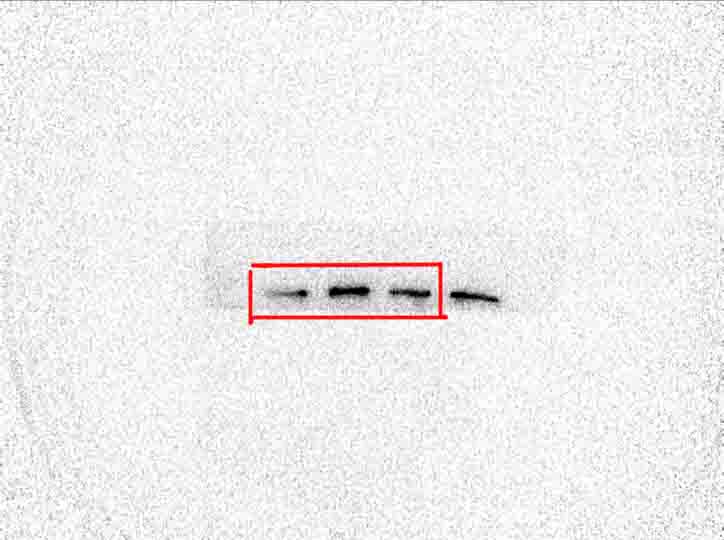

Supplement: Supplementary file 1 [file Data_Sheet_1.ZIP › Original files/original gels/original gels/SW1990 N-CA big picture - required.jpg]

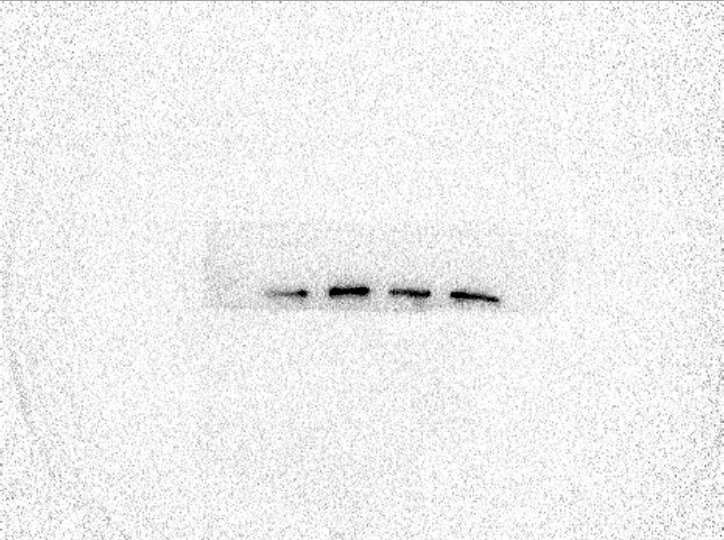

Supplement: Supplementary file 1 [file Data_Sheet_1.ZIP › Original files/original gels/original gels/SW1990 N-CA big picture.jpg]

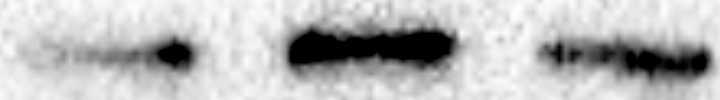

Supplement: Supplementary file 1 [file Data_Sheet_1.ZIP › Original files/original gels/original gels/SW1990 N-CA.jpg]

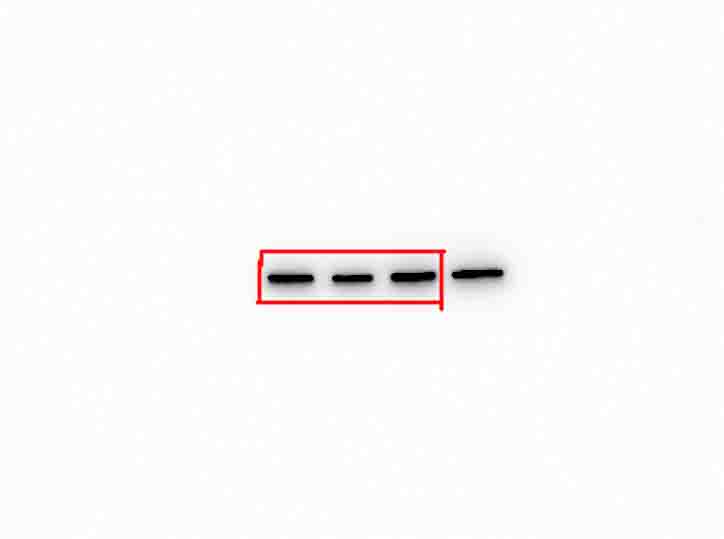

Supplement: Supplementary file 1 [file Data_Sheet_1.ZIP › Original files/original gels/original gels/SW1990 actin big picture - required.jpg]

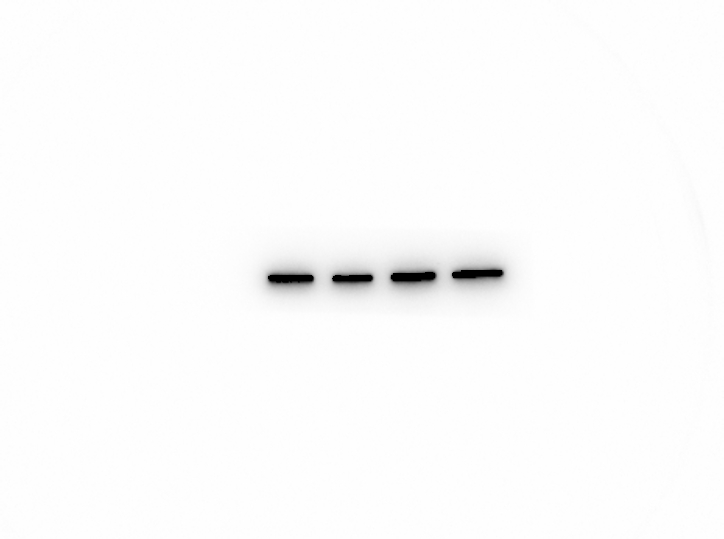

Supplement: Supplementary file 1 [file Data_Sheet_1.ZIP › Original files/original gels/original gels/SW1990 actin big picture.jpg]

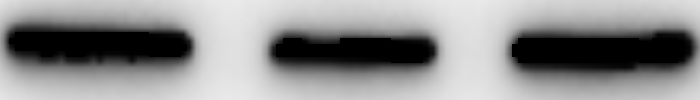

Supplement: Supplementary file 1 [file Data_Sheet_1.ZIP › Original files/original gels/original gels/SW1990 actin.jpg]

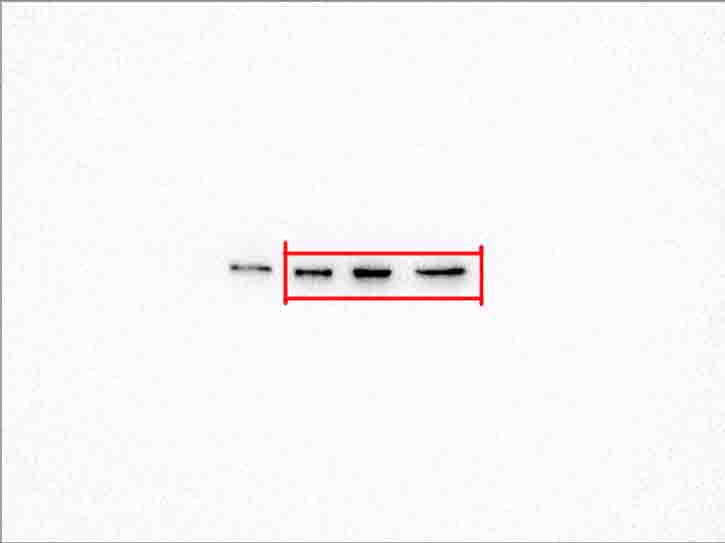

Supplement: Supplementary file 1 [file Data_Sheet_1.ZIP › Original files/original gels/original gels/SW1990 p-GSK big picture - required.jpg]

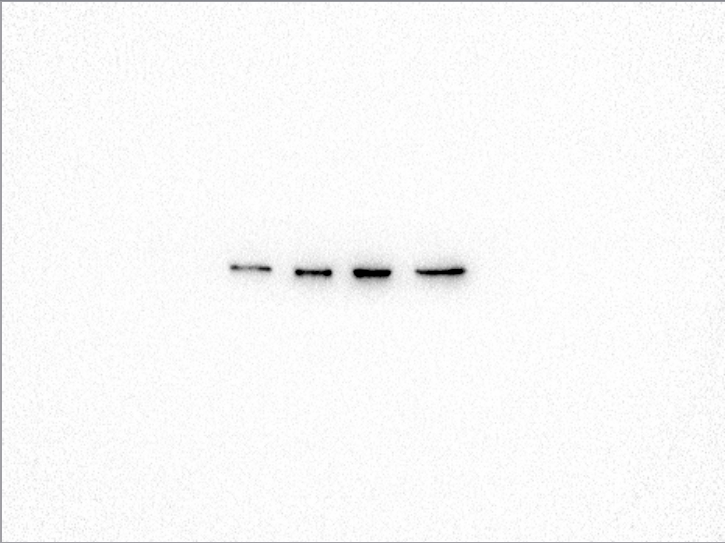

Supplement: Supplementary file 1 [file Data_Sheet_1.ZIP › Original files/original gels/original gels/SW1990 p-GSK big picture.jpg]

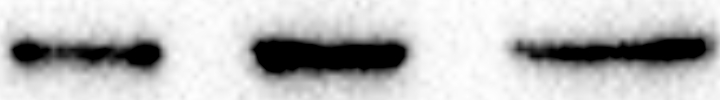

Supplement: Supplementary file 1 [file Data_Sheet_1.ZIP › Original files/original gels/original gels/SW1990 p-GSK.jpg]

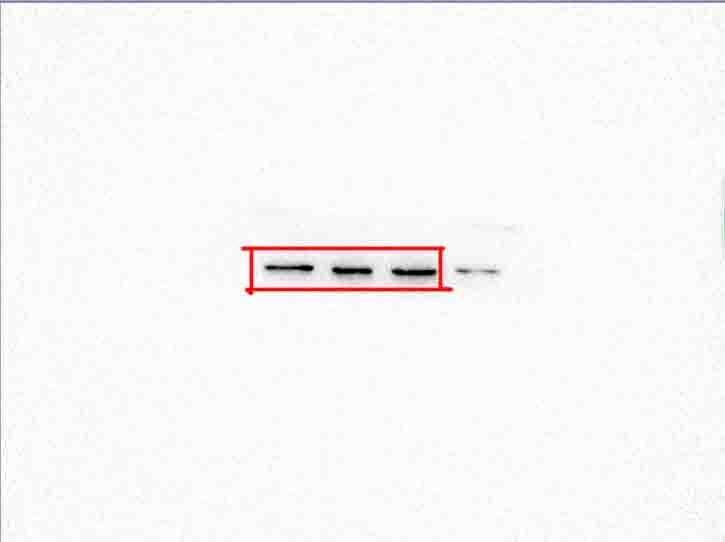

Supplement: Supplementary file 1 [file Data_Sheet_1.ZIP › Original files/original gels/original gels/SW1990 total GSK big picture - required.jpg]

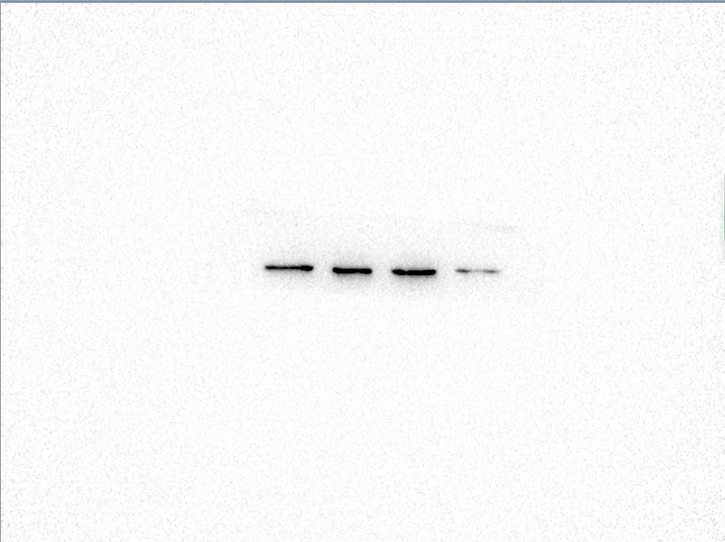

Supplement: Supplementary file 1 [file Data_Sheet_1.ZIP › Original files/original gels/original gels/SW1990 total GSK big picture.jpg]

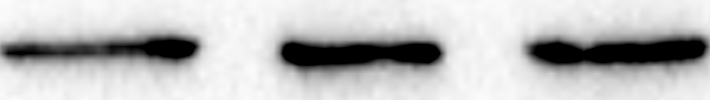

Supplement: Supplementary file 1 [file Data_Sheet_1.ZIP › Original files/original gels/original gels/SW1990 total GSK.jpg]
